# Dual-space visible light authentication toward high security physical unclonable function

**DOI:** 10.1038/s41467-026-75781-4

**Published:** 2026-07-23

**Authors:** Geon Gug Yang, Seong-Gyun Im, Taewoo Kang, Chan Woo Lee, Jonghwa Shin, Seok Joon Kwon, Sang Ouk Kim

**Affiliations:** 1 Department of Materials Science and Engineering, KAIST, Daejeon, Republic of Korea; 2 School of Chemical Engineering, Sungkyunkwan University (SKKU), Suwon, Republic of Korea; 3 KAIST Institute for Nanocentury, KAIST, Daejeon, Republic of Korea; 4 SKKU Institute of Energy Science & Technology (SIEST), SKKU, Suwon, Republic of Korea; 5 Materials Creation, Seoul, Republic of Korea

**Keywords:** Information storage, Self-assembly, Colloids, Photonic crystals, Molecular self-assembly

## Abstract

Random pattern generation by nondeterministic self-assembly offers physical unclonable function (PUF) for hardware-based information security system. However, authenticating intricate signals from randomized disordered structures is challenging without time-consuming, expensive characterization. Here, we present easily authenticatable, highly secure self-assembly-based PUF system enabled by synergistic authentication mechanism exploiting dual-space visible signals. Polycrystalline monolayer morphology of colloidal self-assembled pattern, consisting of a virtual 3D space defined by 2D spatial coordinates and crystal orientation, can be effectively authenticated by combining real-space Bragg reflection and reciprocal-space diffraction signals in visible wavelength regime. Notably, polycrystalline patterned structures composed of hexagonal close-packed colloidal grains are highly beneficial for practical PUF labels, while providing unpredictable reciprocal lattice information with a high degree of freedom for easy authentication with a vast number of encryption keys. Our PUF solely based on structural information can serve for versatile templates for mechanically flexible, renewable, concealable, medium-independent, and biocompatible purposes.

## Abstract (web summary)

Real and reciprocal space visible signals from polycrystalline self-assembly constitute high security optical keys for straightforward authentication that overcomes inherent counterbalance between authentication efficiency against security level.

## Introduction

Gross number of mobile devices and Internet of Things (IoT) is expected to be more than 30 billion by 2030^1,2^. The exponential growth of data/information generation and storage inevitably requires the exigent strengthening of information security. Presently, most of the electronic devices capable of data/information storage rely on the software-based security, such as RSA-2048 protocol, based on the large prime number factorization^3,4^. Such a software-based information security is inherently vulnerable to the brute-force searching with high performance computing hardware, for instance, quantum-computer-based tampering^5,6^. Alternatively, hardware-based media can offer complementary methodology for information security and encryption, while assisting the conventional software-based security system^7^. An ideal hardware-based approach should provide genuine unpredictability arising from the intrinsic randomness in a form of physical unclonable function (PUF), along with the sufficient large degree of freedom to cover a wide range of information security from anticounterfeit to cryptography and authentication^8^. Among various strategies for PUF system, bioinspired principle of self-assembly, which imitates natural self-organization process for random fingerprint wrinkling and neural interconnection, is attracting a great deal of research attentions along with its genuine nondeterministic nature strongly influenced by random thermal fluctuation^9,10^.

Practical PUF labels are expected to meet several figures of merit; (i) extremely high encryption capacity (or information density) with unique responses to multi-stimuli based on nondeterministic structure formation, (ii) easy and fast authentication utilizing typical challenge-response protocol with inexpensive tools, and (iii) cost-effective mass-production to afford the tremendous number of uses (i.e., at least a few hundred of billion). In this regard, optical PUFs are desirable candidates principally owing to their generic inexpensive production as well as easy straightforward authentication with visible spectrum^11^. An ideal optical PUF system requires a judicious balance between maximizing pattern randomness for high security and ensuring straightforward swift authentication, as the information density extractable from an optical image is inherently governed by the resolution characteristics of imaging systems or detectors, such as CMOS sensors. To circumvent this constraint and further enhance the information density, multiple optical response mechanisms, such as Raman scattering, luminescence, polarized responses, or UV–vis absorption spectroscopy, can be integrated^9,12–22^. For an easily accessible authentication, by contrast, many different approaches have explored the use of facile challenge-response pair, such as random scattering patterns from laser irradiation^23–25^, random fluorescent image^26,27^, and reflection signals from structural color^28,29^. For example, 3D assembled colloid structure with local interparticle distance difference may lead to randomized photonic color distribution across the PUF label, enabling a facile authenticable high encoding capacity label^29^. While these approaches have successfully established high-resolution optical authentication mechanisms with well-defined encryption schemes, further research efforts aiming at multi-level high security as well as convenient authentication without additional experimental setups are highly demanded for practical robust security system.

**Fig. 1:**
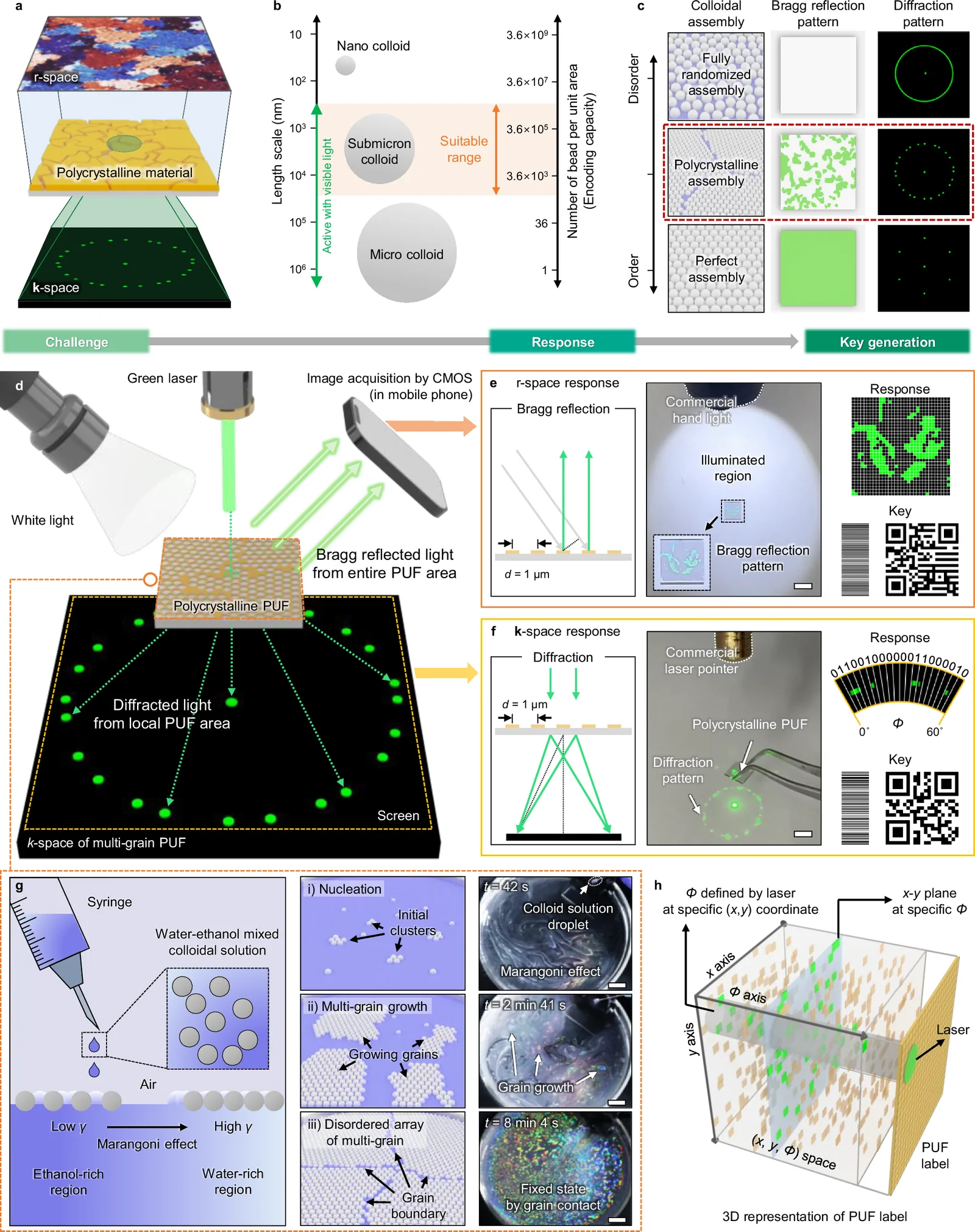
Principle of dual-space multi-grain PUF. **a** Real-space (r-space) and reciprocal-space (**k**-space) representations of polycrystalline materials. **b** Suitable range of colloid sizes based on visible wavelength response. **c** Schematics of fully randomized, polycrystalline, and monocrystalline colloid assemblies, with corresponding Bragg reflection and diffraction patterns. **d** Schematic illustration of the challenge-response-based key generation process using commercial white light and a green laser. **e** Photograph of the Bragg reflection pattern of the PUF and a brief illustration of the key generation mechanism. *d* indicates the periodicity of nanoscale assembled pattern. Scale bars, 1 cm. **f** Photograph of the diffraction pattern of the PUF and a brief illustration of the key generation mechanism. Scale bars, 1 cm. **g** Self-assembly mechanism of polycrystalline colloidal structure with photographs of each step: (i) nucleation of colloids, (ii) simultaneous grain growth at random spots, and (iii) stabilization due to grain contact. Scale bars, 1 cm. **h** 3D space mapping of the PUF label.

Here, we present dual-space responses of monolayer colloidal self-assembled patterns for simple, fast, renewable, and fundamentally unpredictable optical PUF system based purely on the visible spectrum, enabling practical authentication. Our self-assembled PUF tag consists of a virtual 3D space including 2D spatial coordinates in the real space and the crystal orientation at each spatial coordinate. Dual-space response pair can be obtained by the independent manipulation of spatial coordinate and crystalline orientation from a single self-assembled PUF pattern, while using inexpensive unified readout system. Reciprocal lattice information can be directly visualized through light diffraction, whereas real-space information is derived by Bragg reflection, enabling the full retrieval of information from the virtual 3D space to generate a vast number of encryption key combinations. Significantly, the distinct optical responses purely originate from polycrystalline multi-grain structure of monolayer colloidal assembly, which contrasts with the 3D colloidal system exploiting photonic stopband (Supplementary Fig. 1). Combination of the two independent signals from real and reciprocal space (Fig. 1a)^30^ provides a sufficiently large number of key cases (over ~10^184^, 10^20^ from real space 10^164^ from reciprocal space) far beyond the level of single-space-based counterparts. Structural features of the colloidal assembly can be readily transferred to various functional materials, resulting in highly stable multifunctional PUFs. Noticeably, this approach exploiting independent dual-space responses suggests a generic design principle for multi-scale patterned systems with different characteristic feature sizes, such as block copolymer self-assembly, liquid crystals, and nanoparticle assemblies.

## Results

### Authentication and formation principle of multi-grain PUF

We prepared 2D self-assembled monolayer of monodisperse colloidal beads with the unpredictable distributions of polycrystalline multi-grain morphology, whose structural feature could be easily transferred to other materials, such as thin Cr layer with high thermal and chemical stability along with enhanced optical response (Supplementary Fig. 2). Commercially available monodisperse polystyrene (PS) beads with submicron size (532 nm to a few μm) were chosen to harness the optical responses from visible light sources (Fig. 1b). Notably, polycrystalline monolayer self-assembled morphology provides unique real-space (i.e., r-space) and reciprocal-space (i.e., **k**-space) patterns (Fig. 1c)^31^. Perfectly ordered monocrystalline 2D array would show simple plane color in r-space and well-defined 6-fold symmetric diffracted spots in **k**-space^30,32^. Whereas, randomized disordered assembly should lead to a uniform plane image without constructive interference in r-space, while diffuse ring pattern in its **k**-space^31^. Those structures with low degrees of freedom are generically inadequate for high security PUF labels. By contrast, polycrystalline structures with the different orientations and sizes/shapes of self-assembled grains generate nondeterministic distribution of reflected color information in r-space, while giving rise to the randomized distribution of diffraction signals in the **k**-space.

For the straightforward optical authentication of PUF labels under common visible spectrum, conventional white-color LED hand light and green laser pointer (*λ* = 532 nm) sources are used in this work for the reflectance and diffraction authentication, respectively (Fig. 1d). The Bragg reflection of large-area white light from PUF label constitutes a unique optical image arising from the specific constructive interference in the r-space, which is readily convertible to 1D or 2D digital bit arrays, generating up to 10^20^ distinct key configurations with the control of incident light angles (Fig. 1e). The diffraction from the penetrating green laser beam provides a unique circular discrete bit string in **k**-space, readily convertible to an 1D array (Fig. 1f). The independence of two signals from the respective spaces can be combined to form a pair for information cryptography (double-step encryption) as well as one-way single-step information hashing. Furthermore, the diffraction signals can be obtained from different spatial coordinates in a PUF label, while allowing a vast amount of permutation combination to generate the number of different keys up to 10^164^, as enabled by the sequential laser scanning of local areas.

The degree of ordering in the 2D polycrystalline array is basically controlled by manipulating the kinetics of colloidal self-assembly process, as governed by the Marangoni effects at air-water interface (Fig. 1g and Movie S1)^33^. The injection speed of colloidal dispersion could be controlled onto the air-water interface as well as the surface tension difference in the mixed-solvent system (ethanol in water). The resultant interfacial assembly can induce the hexagonal close packing among the neighboring PS colloids such that the degree of ordering is determined by the size and orientation of self-assembled crystalline grains consisting of hexagonal colloid arrays, accompanied by the grain boundaries among them (Supplementary Fig. 3)^34,35^. The formation mechanism of 2D monolayer reveals three distinctive steps. Initially, each PS colloid migrates to the water-rich (high surface tension) portion of air-water interface to nucleate the self-assembled clusters with the randomized orientation and size of the crystalline structure (Fig. 1g. i). Further injection of colloidal dispersion leads to the secondary grain growth from each nucleated cluster (Fig. 1g. ii). Finally, each grain is sufficiently grown-up to meet each other, resulting in a large-area well-packed colloidal monolayer (Fig. 1g. iii). It is noteworthy that Marangoni driving force from the large surface tension difference between evaporating ethanol and water is strong enough to induce the close-packed hexagonal ordering of monodisperse colloids with long-range ordering in the nucleating and secondary growth stages. The typical polycrystalline monolayers can be easily obtained by the rapid spreading of PS colloid dispersion across the entire bath while deploying a high mounting angle of the droplet sliding glass.

Our PUF label can be mapped into a virtual 3D space with the axes composed of the 2D spatial coordinates *x*, *y* for hexagonal unit cell, and the self-assembled crystalline lattice orientation angle, *Φ* at each spatial coordinate (Fig. 1h). In this 3D space, dual space can be designated from r- and **k**-space, corresponding to the optical reflectance and diffraction, respectively. For the r-space encryption, *Φ* value can be controlled as a variable for the fixed *x*, *y* values (i.e., entire PUF label area), while slicing 3D space with an *x*, *y* parallel plane at a specific *Φ* value. Thus, when a specific *Φ* value is given as a challenge, the *x*, *y* coordinates react with the light read-out as the response. For **k**-space encryption, *x*, *y* coordinates can be controlled as variables, and the accumulation of *Φ* values within the laser illuminated area appears as the response. Herein, the 3D space is cut by a *Φ*-axis parallel cylinder with the areal size of illuminating laser beam.

When employing dual-space encryption, we utilize only small portions of information from the *Φ* coordinates for r-space authentication and the *x*, *y* coordinates for **k**-space within a vast three-dimensional space. Consequently, it is difficult to derive a key from one space from the other. Specifically, while trying to tamper from r-space to **k**-space, only a few *Φ* values are chosen as challenge, leaving the remaining *x*, *y* coordinates unexposed and their *Φ* concealed. From the tiny fraction of r-space information, only a few locations of the diffracted angles make it nearly impossible to determine other diffraction information, including the entire distribution of spatial locations and the relative intensities of diffracted beams (Supplementary Fig. 4). Conversely, when tampering from **k**-space to r-space, only a few *x*, *y* coordinates are chosen as challenges, making it impossible to find the *Φ* values of the unexposed area. Besides, the diffraction pattern is simply composed of the relative portion of each different crystalline orientation within the laser illuminated area, without the detailed positional information of each crystalline orientation in the r-space (Supplementary Fig. 5). Furthermore, even when certain optical responses from both r-space and **k**-space are tampered, reconstructing the original nanoscale configuration of PUF pattern is practically impossible without the entire optical responses from all the 3D authentication parameters (Supplementary Fig. 6). Moreover, the detailed formation for structural defects in the original polycrystalline self-assembled morphology, such as dislocations and point defects, cannot be retrieved from the optical responses. Overall, although our dual-space responses share a tiny amount of information arising from the minor overlap of information within 3D virtual space, they can still be considered as independent modes for encryption.

### Nondeterministic Bragg reflection pattern-based key generation

**Fig. 2:**
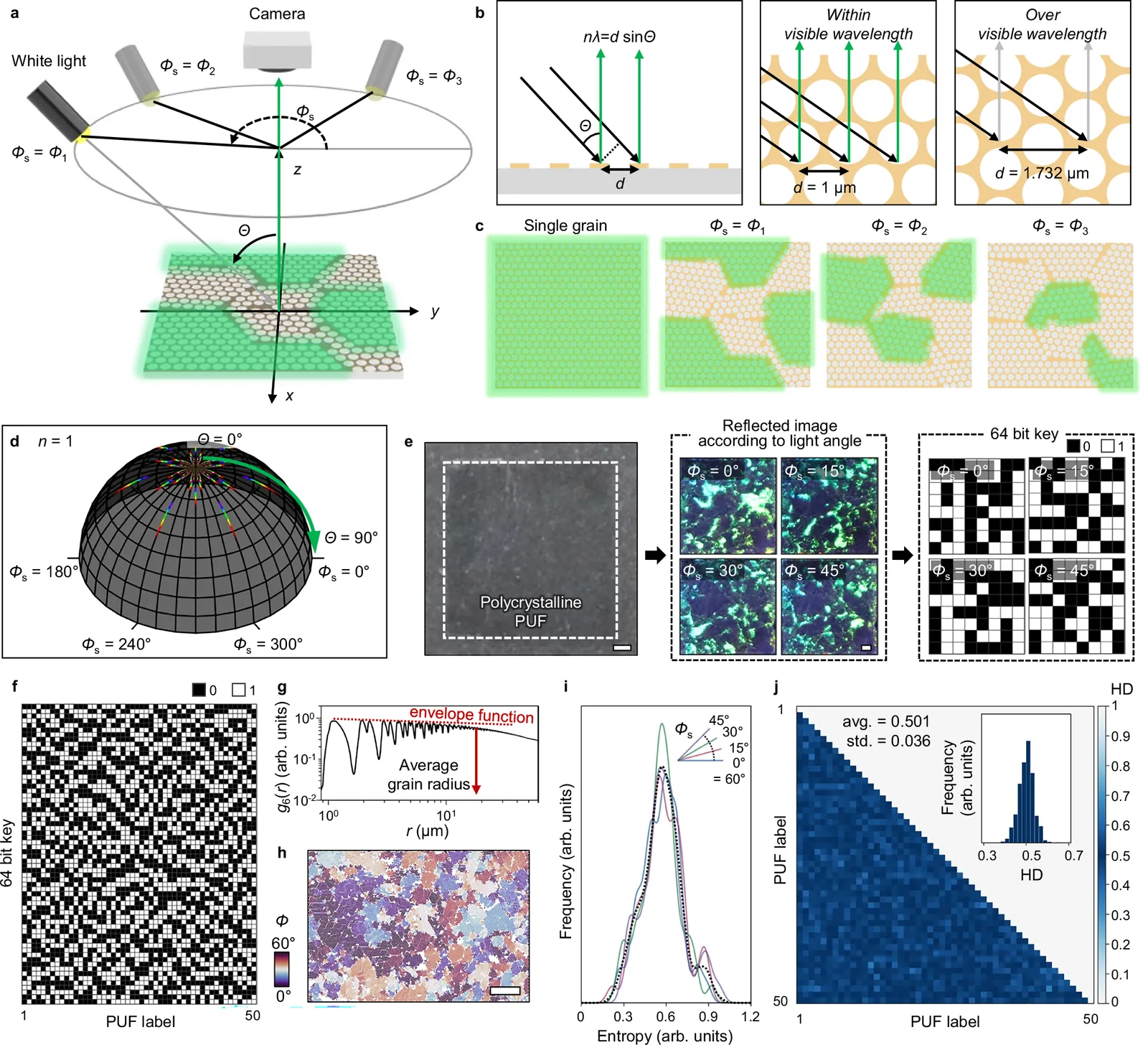
Nondeterministic Bragg reflection pattern-based unique key generation. **a** Schematic illustration of response generation based on Bragg reflection patterns. **b** Schematic illustration of the mechanism for generating area-selective Bragg reflection patterns. **c** Schematic illustration of various Bragg reflection patterns depending on the direction of white light. **d** Simulation results of localized constructive interference colors (color: reflected wavelength) as a function of the incident light direction (*θ*, *Φ*_S_) for first-order diffraction (*n* = 1). **e** Key generation flow based on the Bragg reflection pattern of the PUF. Scale bars, 1 mm. **f** 64-bit key array for 50 different PUFs at a specific *Φ* value. **g** Calculation of the average grain radius based on SEM images of the PUF. (*g*_6_(*r*): bond-orientational correlation function, log-log scale). **h** Orientation map of the PUF label. Scale bars, 25 µm. **i** Probability density distribution of entropy as a function of the direction of white light. **j** Hamming distance (HD) calculation of Bragg reflection pattern-based keys from 50 different PUF labels.

The unique Bragg reflection image in r-space originates from the area-selective constructive interference reflected from polycrystalline colloidal morphology^36,37^. The visible image resulting from constructive interference is determined by the incident light direction against the crystalline lattice orientation, as defined by colatitudinal (or polar) angle (*θ*) and longitudinal (or azimuthal) angle (*Φ*_S_) (Fig. 2a). While the reflectance color is determined by *θ* (Supplementary Fig. 7), the local occurrence of Bragg reflection is governed by *Φ*_S_ (Supplementary Fig. 8), which defines the relative orientation between incoming light and self-assembled crystalline lattice. As illustrated in Fig. 2b, c, the polycrystalline nature of 2D monolayer assembly leads to the randomized distribution of *Φ* for different crystalline grains under the fixed incident light condition and thereby produces different reflectance for each grain. Accordingly, the *Φ*_S_ can serve as a challenge in the r-space. Our simulation (Fig. 2d and Supplementary Fig. 9) illustrates the localized constructive interference, where the light irradiation angle (*Φ*_S_) and crystal orientation (*Φ*) are parallel to each other. We choose a specific *θ* for the constructive interference of green light for an easy visibility. The Bragg reflection pattern is unpredictably changed within the range of 0° ≤ *Φ*_S_ ≤ 60° (due to 6-fold symmetry), resulting in the challenge-response pairs. The uniqueness of challenge-response pair can be tested for the four different *Φ*_S_ values (0°, 15°, 30°, and 45°) to generate 50 different PUF labels (totally 200 responses) (Supplementary Fig. 10). Given a specific *θ*, different *Φ*_S_ generates different reflectance image, as shown in Fig. 2e. After extracting the key array from a binary converted Bragg reflection image, Neumann bit debiasing algorithm was employed to generate the unique key array for each *Φ*_S,_ with the bit uniformity converging to the ideal value of 0.5 (Fig. 2f and Supplementary Fig. 11 and 12)^23,38^.

The size, orientation, and the number of crystalline grains are the crucial parameters in dealing with the optical reflectance. From a myriad of grains with randomized orientations, the average grain size can be determined by the exponential decay of orientation correlation, which corresponds to ~20 μm for the typical condition with 1:1 ethanol/water mixture and 40° mounting of droplet sliding glass (Fig. 2g). Considering the average grain size, the 1 × 1 cm^2^ PUF label contains ~80,000 grains, which is large enough to generate a myriad of different Bragg reflection patterns (Fig. 2h). The intrinsic complexity in each response can be quantized by calculating the entropy of response^9^. We used permutation entropy^39^, generically adequate to measure the complexity of noisy random pattern. As shown in Fig. 2i and Supplementary Fig. 13, the probability density distribution of permutation entropy is constant regardless of *Φ*_S_ (avg = 0.5373, std = 0.1263). This verifies that the intrinsic complexity stemming from polycrystalline nature is not biased for any specific *Φ*_S_ but uniformly distributed across all directions with a sufficiently high level. Such an intrinsic complexity is sufficient for the randomization of information security uniqueness. To verify the uniqueness of key array based on our Bragg reflection pattern, inter-Hamming distance (HD) was calculated from multiple PUF labels against a single challenge (Fig. 2j and Supplementary Fig. 14), which yields nearly ideal value of 0.501 ± 0.036. Furthermore, intra-HD analysis was performed to assess the reproducibility and consistency of responses from the same PUF label, yielding the value of 0.007 ± 0.005 (Supplementary Fig. 15). Therefore, our optical Bragg reflection PUF in the r-space offers nearly ideal and unique complexity to be used in the information encryption. As typical examples, this Bragg reflection-based mode can be readily applied to practical items, such as valuable original paintings and premium liquors, to distinguish them from counterfeits (Supplementary Fig. 16).

### Nondeterministic diffraction pattern-based key generation

**Fig. 3:**
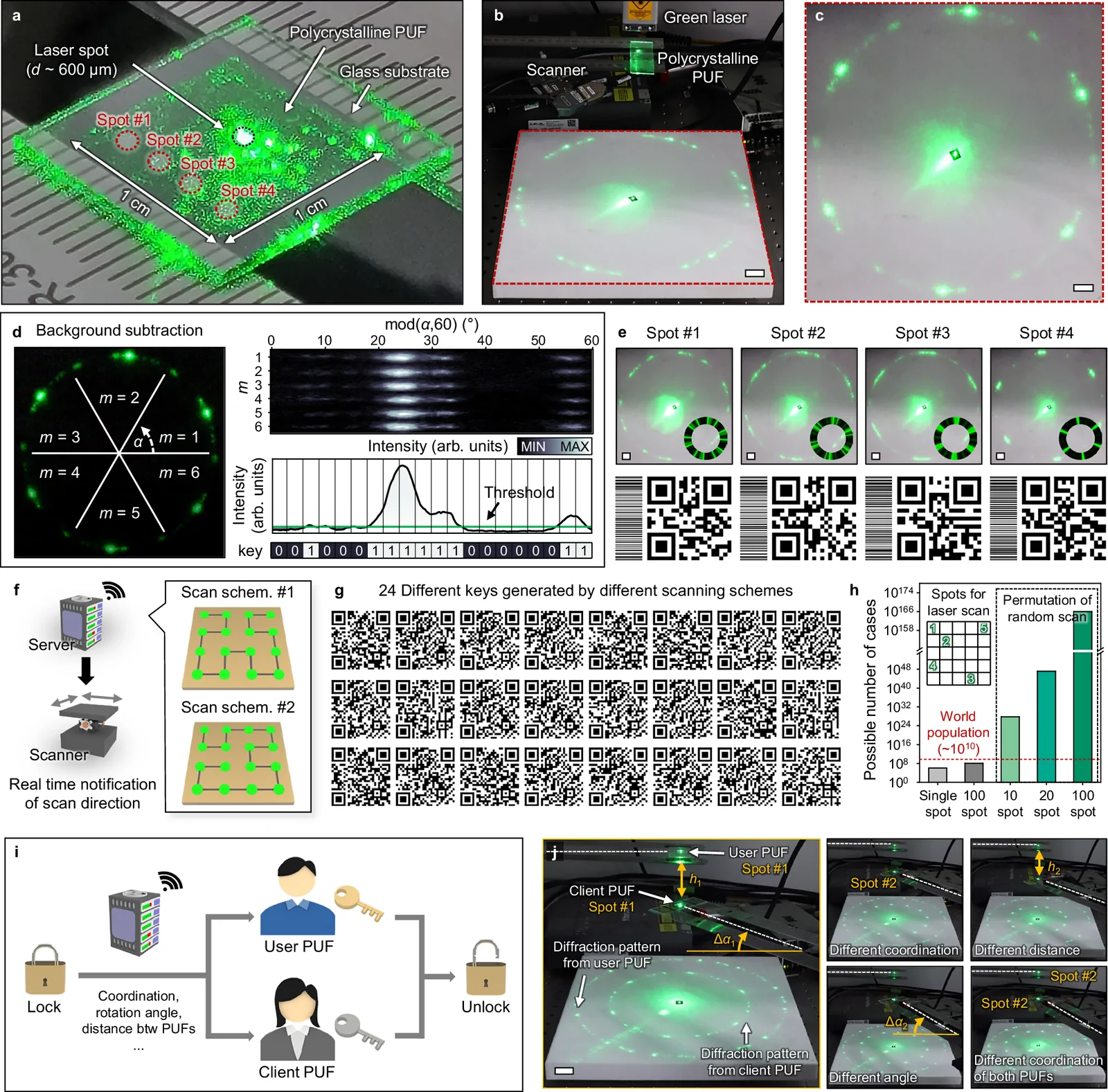
Nondeterministic diffraction pattern-based key generation. **a** Photograph of a 600 μm green laser on a 1 × 1 cm² PUF label. **b** Homemade laser scanning system for generating diffraction pattern-based responses. Scale bar, 2 cm. **c** Photograph of the diffraction pattern from a local PUF area. Scale bar, 2 cm **d**, Background-subtracted image of the diffraction pattern and 1D bit-array extraction along the azimuthal angle *α*. **e** Diffraction patterns from different spots along with corresponding barcode and QR code images. Scale bars, 2 cm. **f** Schematic illustration of real-time notification of scan direction to generate different keys from the same PUF label. **g** Examples of different QR code generations from the same PUF label using laser scanning. **h** Calculated number of possible cases from combining keys of multiple spots (grey) and from permutations of randomly scanned spots (green), compared with the world population (~10^10^). **i** Schematic illustration of a dual security system using PUF superposition. **j** Photograph showing possible variations using the superposition of two different PUFs. Scale bar, 2 cm.

Although the optical Bragg reflection PUF may offer satisfactory uniqueness as mentioned above, its intrinsic characteristic based on the r-space optical image can suffer from a variety of tampering or hacking trials for counterfeit, duplication, or manipulation. In this regard, we introduce **k**-space response by utilizing the diffraction pattern from the monochromatic green laser transmitting through PUF labels (*λ* = 532 nm, beam diameter 600 µm) (Fig. 3a). Notably, r- and **k**-spaces should possess mathematically equivalent information for a given volume of media. Nevertheless, the **k**-space response from the entire PUF area with polycrystalline nature typically leads to a simple circular pattern, which is hardly useful for a nondeterministic PUF application. For the minimal correlation between r- and **k**-space responses, we employ a smaller sampling area for the **k**-space responses, as defined by a typical laser beam size. For example, while using 600 μm diameter laser beam within 1 × 1 cm^2^ PUF label area (spot area), more than 100 different diffraction patterns can be obtained by scanning over the entire sample areas. The small laser beam area results in the localized information for the **k**-space (Fig. 3b, c), while the white light illumination over the entire PUF area exhibits the global information in the r-space (Bragg reflection images). Laser illumination at a specific location generates unique diffraction pattern, which can be processed to be 1D digital string with the designated resolution angle in **k**-space (Fig. 3d). Notably, the diffraction pattern can be reliably extracted regardless of surrounding environmental condition with a proper background image normalization method (Supplementary Fig. 17). The variation of laser illumination location on the PUF area leads to different and unique signals (Fig. 3e). Assuredly, the small, localized area for obtaining **k**-space response is associated with the limited size of information (typically about 2^20^ (~10^6^) for 600 μm diameter laser beam); nonetheless, the sequential scanning of 100 different spots can be permuted (100! ~ 10^158^), which in turn generates a myriad of local information combinations. For example, with the 100 permutations of local responses containing 20 bits for each will generate 10^164^ different cases in total.

Owing to the unpredictability for a specific series of scanning sequence out of the permutation, r- and **k**-space responses are not correlated, and therefore, cannot be inferred from one another. For permutation, the sequential order of laser scan can be real-time notified by server and precisely controlled by a programmable XY stage (Fig. 3f and Movie S2). Therefore, a single cycle of sequential laser scanning and its random permutation is fast enough to generate unique and unpredictable **k**-space responses. The fast and precise control in the optical PUF in **k**-space will generate a vast amount of randomizable information, as shown in the exemplary 2D QR images in Fig. 3g. Indeed, the possible number of cases grows exponentially with the number of scanning locations (Fig. 3h). In particular, the permutation with only the 10 locations of laser illumination will generate the number of keys exceeding the total expected number of IoT devices and mobile devices (up to 30 billion by the year 2030)^1,2^. Therefore, the optical PUF in **k**-space authentication provides unique and sufficiently large randomizable information.

Unlike r-space authentication based on surface Bragg reflection, **k**-space characterization employing the laser light penetrating through PUF label allows a straightforward superposition of multiple PUF labels (i.e., user PUF and client PUF) for the further increase of information security (Fig. 3i). Even under the potential tampering of user PUF by an external attack, the extraction of information can be thwarted, while securing the hidden information in the client key. The two superposed different PUF labels generate two concentric diffraction rings, as shown in Fig. 3j. Indeed, the relative angle of label orientation can also be arbitrarily controlled for further enlargement of possible information. Thereby, such a superposition ensures the complete independence of the r-space information solely based on the user PUF revealed at the top surface from the **k**-space information combining the two PUF labels in different layers.

For a reliable **k**-space-based authentication for arbitrary end user, 3D printed user-kit was prototyped with an easily aligning system. The kit consists of a diffraction image plane with 50 coordinate holes, a four-legged structure for fixing PUF label at the desired coordinate, and a laser pointer holder (Supplementary Fig. 18). Different designated coordinates can be readily aligned by user, enabling a reliable generation of diffraction pattern. In order to verify the reproducibility of pattern generation, multiple users were aligned with the same coordinate (Supplementary Fig. 19). For all users, the exact same diffraction pattern could be regenerated in a repeated way, confirming the reliable and repeatable pattern generation for practical applications. Notably, after a careful evaluation of the overall inexpensive fabrication costs for PUF label and user kit, practical advantages of our PUF system for real-world scenarios could be highlighted (Supplementary Table. 1).

### Randomness and uniqueness of optical PUF in k-space

**Fig. 4:**
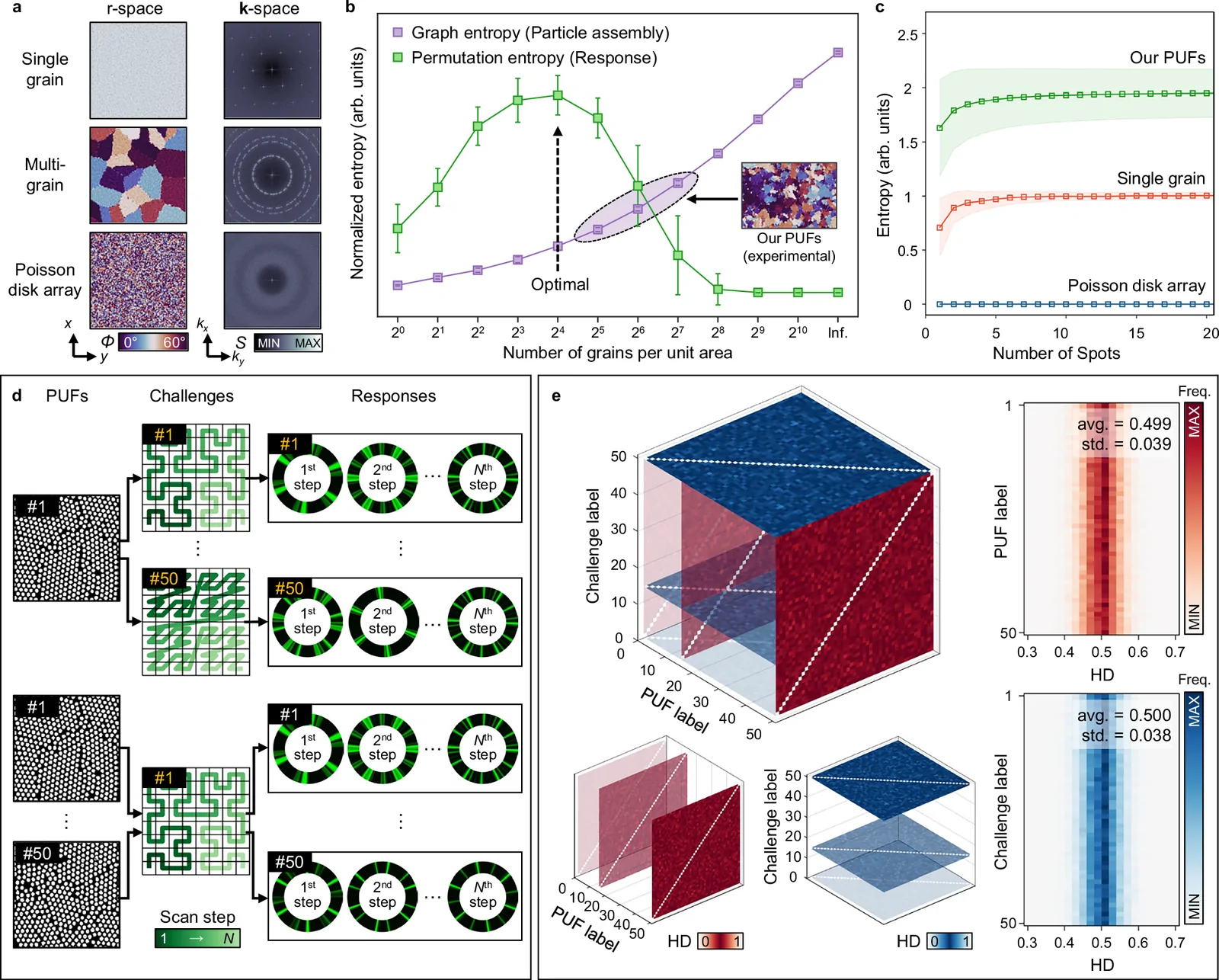
Randomness and uniqueness of diffraction pattern-based PUFs. **a** Simulation results for r-space and **k**-space patterns of 2D single grain, multi-grain, and Poisson disk array. **b** Permutation entropy and graph entropy calculations based on the number of grains per unit area. Data are presented as mean values ± standard deviation. **c** Calculated entropy for single grain, Poisson disk array, and the present PUF as a function of permutation spot number. Data are presented as mean values ± standard deviation. **d** Schematic illustration of uniqueness tests for multi-PUFs with a single challenge pair (top) and a single PUF with multiple challenge pairs (bottom). **e** 3D mapping of calculated HD for multi-PUFs with a single challenge pair (red) and single PUF with multiple challenge pairs (blue).

For the evaluation of uniqueness and quality of the information generated by our PUF label, particularly in **k**-space, we have calculated the complexity of diffraction pattern. Similar to r-space, the monolayer colloidal assembly with polycrystalline nature is crucial for a sufficiently randomizable information in the **k**-space. The perfectly ordered or fully disordered array leads to nearly null information (Fig. 4a). The correlation between the intrinsic complexity of a multi-grain PS bead array and its **k**-space response can be illustrated by the simulation of multi-grain growth of a Poisson disk array^40^. The intrinsic morphological complexity (i.e., graph configurational entropy) and the **k**-space complexity (i.e., permutation entropy) could be calculated for the different numbers of grains per unit area^41,42^. Figure 4b presents that the **k**-space pattern complexity is maximized when the number density of grains is in the range of 2^3^ to 2^5^. Experimental measurement of the entropy obtained from the randomly acquired SEM images of colloidal assembly also confirms the highest complexity of **k**-space pattern in the same range of grain density. As shown in Fig. 4c, polycrystalline pattern can exhibit a much higher entropy as a function of the number of laser scans for permutation. The uniqueness of diffraction pattern can be checked by HD characterization for the random permutated combinations of 50 different diffraction patterns, where the permutation order works as a challenge (Fig. 4d, detailed in method). A single PUF subjected to multiple challenges, as well as multiple PUFs to single challenge (inter-HD), commonly exhibits nearly ideal HD values of 0.499 ± 0.039 and 0.500 ± 0.038, respectively (Fig. 4e and Supplementary Fig. 20 and 21). Besides, intra-HD analysis for multiple identical challenges yields values of 0.011 ± 0.008 (Supplementary Fig. 22), confirming the consistent production of similar keys under the same conditions. This successfully confirms the uniqueness and robustness as well as intrinsic randomness of our optical PUF in the **k**-space.

### Multifunctional PUF labels for various purposes

**Fig. 5:**
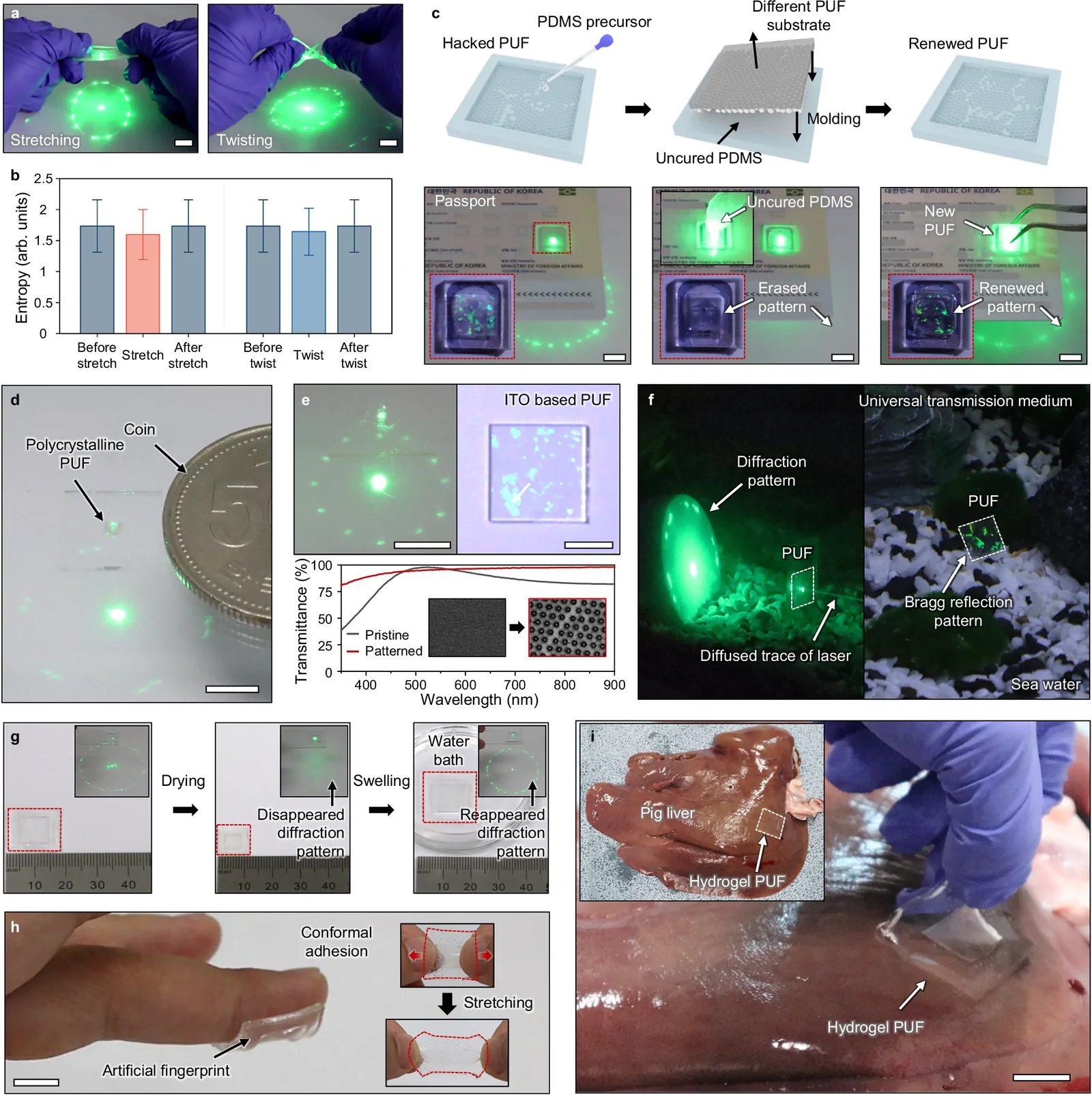
Multifunctional PUF labels for various applications. **a** Photograph of stretching and twisting tests on a PDMS-based elastic PUF. Scale bars, 1 cm. **b** Information entropy calculations before, during, and after mechanical stress. Data are presented as mean values ± standard deviation. **c** Schematic illustration of a renewable PUF with a demonstration photograph on a passport. Scale bars, 1 cm. **d** Diffraction pattern image of a hidden PUF. Scale bars, 5 mm. **e** Diffraction and Bragg reflection patterns of ITO-based PUF, along with their UV-vis spectroscopy profiles. Scale bars, 5 mm. **f** Underwater authentication capability of PUF in a deep-sea-like environment. **g** Reversible diffraction pattern generation of hydrogel PUF after drying and re-swelling. **h** Artificial fingerprint demonstration using hydrogel PUF. Scale bars, 1 cm. **i** Bio-compatible hydrogel PUF demonstrated on pig liver. Scale bars, 1 cm.

We examined the diverse functionality, reusability, and even renewability of our dual-space PUF, while employing versatile material compositions ranging from transparent elastomers or highly stable metals or inorganic transparent conductors to hydrogels. The self-assembled colloidal polymer pattern can serve as a template for the subsequent pattern transfer towards the various types of designated materials. We first produced thin elastomeric media (i.e., PDMS) with 2D inverse patterns (Fig. 5a)^43^. The PDMS pattern replicates a colloidal self-assembled structure by simple replica molding, which maintains similar diffraction patterns even under the diverse modes of mechanical deformations (i.e., bending, stretching, or twisting). Noticeably, the original level of information entropy in an undeformed PUF label can be readily restored after releasing the mechanical stress (Fig. 5b).

From the practical point of view, a PUF label implanted on a target device structure is difficult to replace, while concerning the potential damage to the sophisticated functional device. Accordingly, an easily renewable PUF label is highly demanded for the practical features, such as reusing hacked devices. Suppose a specific PUF label is tampered, the original PUF pattern in the elastomeric media can be simply erased by applying additional PDMS precursors (Fig. 5c). Subsequently, a new different PUF pattern can be straightforwardly engraved at the additional PDMS layer by replica molding. Therefore, our PUF label can be easily reused, recycled, and renewed on demand. In the exemplary test for the identity authentication in a passport, the tampered original PUF labels can be easily replaced with the renewed PUF labels, while securing the equivalent level of information complexity.

Ironically, easy duplication of PUF pattern by replication molding can be misused for fake authentication. In this regard, original colloidal pattern could be pattern transferred to thin metal layer. While using an ultrathin metal layer thickness (For example, 3 nm for Cr), it is difficult to replicate the Cr pattern by simple replica molding (Supplementary Fig. 23). Nonetheless, a sufficiently high contrast in the refractive indices can be secured between metallic layer and the surrounding empty space to yield clear diffraction and Bragg reflection patterns. Additionally, as mentioned above, the metal pattern may offer greatly enhanced thermal and chemical stability compared to the original polymeric pattern (Supplementary Fig. 24 and 25). Besides, information security of the original colloidal pattern or any replica pattern could also be protected by a transparent encapsulation layer. Mechanically and chemically stable, transparent Parylene layers could be conformally deposited on the PUF labels to avoid the direct replica molding of PUF pattern (Supplementary Fig. 26). While the Parylene layer effectively encapsulates the entire area of PUF pattern, the real and reciprocal optical responses were successfully regenerated without any considerable disruption.

Our PUF labels can also be miniaturized and transferred to optically transparent materials, facilitating the security purposes as hidden PUFs. A PUF label with the lateral size comparable to an external laser illumination size (few hundred microns) can be implemented in a hidden tiny area of target system (Fig. 5d). Moreover, PUF pattern can be transferred to an indium tin oxide (ITO) surface by sputtering onto the 2D colloidal assembly layer^44^. The ITO with inverse PUF pattern is optically transparent and electrically conductive, and therefore, can be applied to various optical and optoelectronic parts and devices with the generic information security functions installed (Fig. 5e and Supplementary Fig. 27). Besides, since our PUF pattern is located on the surface of a reflective inorganic material, the reflected diffraction pattern can then be used for authentication (Supplementary Fig. 28). Our PUF labels can also be authenticated under the unusual environments, such as in the dark seawater, without requiring specialized equipment. A reliable operation could be verified with typical green laser and hand light illumination for the r-space and **k**-space response (Fig. 5f).

The potential use of PUF label could also be examined in biomedical areas, while employing a hydrogel structure, well-known for its compatibility with human skin and organs^45,46^. Hydrogel film containing inverse PUF pattern could be prepared by a similar replica molding process with the PDMS PUF preparation. Notably, the characteristic length scales of hydrogel PUF are controllable with the level of water swelling. A severe evaporation of water may distort the original polycrystalline structure, yielding a faint diffraction in the **k**-space (Fig. 5g). However, the original PUF pattern can be readily restored by water swelling again. Besides, the hydrogel PUF labels are advantageous for the biocompatible purposes, such as wearable or even bio-implantable artificial security devices. Hydrogel PUF labels can be conformally stabilized on human skin and organ surfaces (Fig. 5h). Accordingly, they can be used for the unique identity and information security towards, for instance, transplant organs (Fig. 5i). Finally, for the functional validation of PUF labels composed of diverse materials, representative PDMS- and ITO-based PUF arrays were fabricated (Supplementary Fig. 29) and their intra- and inter-HD values were compared, similar to the analysis in Fig. 4. Regardless of material type, near-ideal values could be obtained (Supplementary Figs. 30 and 31). Notably, encryption keys could be successfully extracted from these multifunctional PUFs with different material characteristics (Supplementary Fig. 32).

## Discussion

Independent dual-space visible wavelength signals generated from polycrystalline self-assembled PUFs have been successfully integrated to constitute a vast library of unique keys, sufficiently covering the enormous growing number of the entire IoT devices over worldwide. Interestingly, polycrystalline multi-scale morphology has been verified more practical than fully randomized disordered assembly for straightforward PUF applications. Polymeric colloidal self-assembled patterns can serve as master templates for versatile ubiquitous PUF labels, widely applicable in various nontypical environments, including marine accidents, organ trafficking, and beyond. It is noteworthy that our visible optical signal-based PUF system is anticipated to be easily integrated with diverse optical computing or communication devices. Beyond the well-established optical PUF mechanism, our dual-space authentication mechanism ensures multi-layer information security as well as accessible unified authentication towards practically meaningful PUF system. Inspired by the natural nondeterministic pattern generation in fingerprint, vein network and others, self-assembly principle intrinsically randomized by ambient thermal fluctuation offers a new prospect for artificial information security.

## Methods

### Materials

PS colloid 10 wt% solution was purchased from Alfa Aesar. Ethanol, sulfuric acid, and hydrogen peroxide were purchased from Sigma-Aldrich. Sylgard 184 and SE1700 were purchased from Dow Corning. Chlorotrimethylsilane, Acrylamide (AAm), sodium alginate, ammonium persulfate, calcium sulfate, methylenebisacrylamide, and *N,N,N*′*,N*′-tetramethylethylenediamine were purchased from Sigma–Aldrich. Fresh pork liver was purchased from a local market (Daejeon, Republic of Korea).

### Fabrication of polycrystalline self-assembly of PS colloid and PUF label

PS colloid powder was obtained by evaporating the water from the PS colloid 10 wt% solution. The PS colloid powder was then dispersed in an ethanol/water (1:1) mixed solvent at a concentration of 10 wt%. A glass slide was treated with piranha solution (Sulfuric acid: Hydrogen peroxide = 7:3) to clean it and enhance its hydrophilicity. The hydrophilic glass slide was mounted on a glass dish at a 40° angle and fixed with carbon tape. A well-dispersed PS colloid solution was contained in a syringe and gently dropped onto the glass slide. The droplet slid to the air-water interface and spread into the water-rich region. After the assembly was completed, the PS colloid pattern was gently transferred to an Eagle-XG glass substrate and fully dried.

### PS colloid pattern transfer to various materials

For the general PUF label, the PS colloid size was reduced by O_2_ plasma treatment to create an intergap between the PS colloids. Cr metal was deposited onto the size-reduced PS colloid pattern, and a lift-off process was conducted to obtain a metal mesh structure. For the indium tin oxide (ITO) PUF label, ITO was deposited onto the size-reduced PS colloid pattern by sputtering, and a lift-off process was conducted to obtain the ITO mesh structure. For the PDMS PUF label, the PS colloid-assembled substrate was treated with UV-ozone (UVO) and exposed to chlorotrimethylsilane to facilitate easy detachment after PDMS curing. PDMS oligomer was mixed with the curing agent in a 10:1 ratio and poured onto the PS colloid-assembled substrate. After sufficient vacuum treatment, PDMS was cured at 60 °C for one day. The PDMS PUF was detached after curing. For the hydrogel PUF label, the PS colloid-assembled substrate was also treated with UVO and exposed to chlorotrimethylsilane to facilitate easy detachment after hydrogel curing. PAAm/alginate gel solution was mixed with APS solution in a 49:1 ratio and poured onto the PS colloid-assembled substrate. During hydrogel curing, the solution was covered with a glass slide. After curing at 60 °C for 30 min, the hydrogel PUF was detached from the substrate.

### Response generation setup

For Bragg reflection-based authentication, a commercial white LED hand light was fixed on a stand at the proper polar angle, and a digital camera or mobile phone was positioned directly above the PUF label to take a snapshot. The hand light or PUF label was rotated to obtain azimuthal angle-dependent Bragg reflection patterns. For diffraction-based authentication, a commercial laser pointer was used for simple demonstration. For the scanning-based diffraction authentication system, a static green laser (532 nm, 56 mW, Golden Light) was fixed on the wall of the optical table, and an X, Y scanner (XMS 100, Newport) was fixed on the optical table.

### Characterization

The morphologies of PUF label were characterized by SEM (Hitachi S-4800). The transmittance of ITO and ITO PUF label was analyzed using UV-vis absorption spectroscopy (Shimadzu, UV-2600).

### Structure factor calculation

The structure factor is defined as the Fourier transform of the point pattern in real space as follows:

1 $${{{\bf{S}}}}\left({{{\bf{k}}}}\right)={\sum}_{n=1}^{N}f\left({{{{\bf{r}}}}}_{n}\right)\exp \left(i{{{\bf{k}}}}\cdot {{{{\bf{r}}}}}_{n}\right)$$

where *N* is the number of coordinates in the system, **r**_*n*_ denotes the *n*-th coordinates in real space, **k** denotes the coordinates in reciprocal space, and *f* represents the scattering intensity.

### Bond-orientational order parameter (BOP) calculation

The orientation of the point array exhibiting the 6-fold symmetry can be characterized by the bond-orientational order parameter (BOP).

2 $${\varPsi }_{6}^{j}=\frac{1}{{n}_{j}}{\sum}_{k=1}^{{n}_{j}}\exp \left(i6{\phi }_{{jk}}\right)=\frac{1}{{n}_{j}}{\sum}_{k=1}^{{n}_{j}}\cos \left(6{\phi }_{{jk}}\right)+i\sin \left(6{\phi }_{{jk}}\right)$$

where *n*_*j*_ is the number of neighboring (i.e., bonded) particles of particle *j*, and *φ*_*jk*_ is the phase angle between *j*-th particle and its *k*-th neighboring particle.

The bond orientational correlation can be defined using BOP as follows:

3 $${g}_{6}\left(r\right)=\frac{{\left\langle {\int }_{r}^{r+\varDelta r}{\sum }_{k\ne j}{{\mathrm{Re}}}\left({\varPsi }_{6}^{j}{\varPsi }_{6}^{k*}\right)\delta \left({r}^{{\prime} }-{r}_{{jk}}\right)d{r}^{{\prime} }\right\rangle }_{j}}{{\left\langle {\int }_{r}^{r+\varDelta r}{\sum }_{k\ne j}\delta \left({r}^{{\prime} }-{r}_{{jk}}\right)d{r}^{{\prime} }\right\rangle }_{j}}$$

where *N* is the dominant symmetry number (i.e., the number of neighboring particles), *Ψ*_6_^*^ is the complex conjugate of *Ψ*_6_, and Re(.) denotes the real part of the complex number.

As the distance *r* increases, bond orientational correlation exhibits three types of decaying behaviors^47^:

4 $${g}_{6}\left(r\right) \sim \left\{\begin{array}{c}C ;\hfill \, {{{\rm{Constant}}}}\,\,\qquad \qquad \\ {r}^{-{\eta }_{6}}; \hfill \qquad{{{\rm{Power}}}}\; {{{\rm{law}}}}\; {{{\rm{decay}}}} \,\,\,\, \\ \exp \left(-\frac{r}{{\xi }_{6}}\right); \qquad{{{\rm{Exponential}}}}\; {{{\rm{decay}}}}\end{array}\right.$$

In well-ordered system exhibiting quasi-long range orientational order, the correlation function manifests a power-law decay with the exponent *η*_*6*_. Conversely, in polycrystalline system, the orientational correlation is maintained within individual grains but suffers abrupt changes at grain boundaries. The average grain size can be estimated by identifying the distance *r* at which the decaying behavior of the correlation function undergoes a significant change.

### LZ entropy calculation

Lempel-Ziv (LZ) complexity is a measure of the complexity of a data sequence, which is based on the analysis of unique patterns within the sequence^48^. The LZ complexity, denoted as *C*_LZ_, is defined by the compressibility of the data, i.e., how much the data can be compressed using the LZ compression algorithm. It has been established that as the size of the data sequence increases, *C*_LZ_ approaches the Kolmogorov complexity, which is the minimum size required to represent the data fully. However, since *C*_LZ_ is limited by being proportional to the length of the sequence, the LZ entropy (*S*_LZ_) was proposed as a normalized measure of *C*_LZ_. The *S*_LZ_ for an *n*-base data with length *N* is defined as follows:

5 $${S}_{{{{\rm{LZ}}}}}=\frac{{C}_{{{{\rm{LZ}}}}} \, {\log }_{n} \, N}{N}$$

### Sample entropy calculation

Sample entropy (*S*_samp_) is another metric used to assess the complexity of a signal by measuring the similarity between subsequences within the input sequence^49^. The input sequence **X** of length *N* is divided by *N* – *m+*1 subsequences (**X**_*m,i*_) with an embedding dimension of *m*. Two subsequences are considered similar if the Chebyshev distance *d*_∞_ between them is less than a specified distance threshold *r*. The similarity between subsequences can be quantified by the correlation integral *C*_*m*,*i*_(*r*), which represents the probability of finding a subsequence similar to the *i*-th subsequence^50^

6 $${d}_{{{\infty }}}\left({{{{\bf{X}}}}}_{m},{{{{\bf{Y}}}}}_{m}\right)={\lim}_{p\to {\infty }}{\left[{\sum}_{k=1}^{m}{\left({X}_{k}-{Y}_{k}\right)}^{p}\right]}^{\frac{1}{p}}={\max }_{k\in \left[1,m\right]}\left|{X}_{k}-{Y}_{k}\right|$$

7 $${C}_{m,i}\left(r\right)=\frac{1}{N-m+1}{\sum}_{j=1}^{N-m+1}u\left(r-{d}_{{{\infty }}}\left({{{{\bf{X}}}}}_{m,i},{{{{\bf{X}}}}}_{m,j}\right)\right)$$

where *u*(.) is the Heaviside function.

With the correlation integral, *S*_samp_ can be calculated by considering the number of similar subsequences for embedding dimensions of *m* and *m* + 1, respectively. This provides a measure of the complexity of the signal, as a higher number of distinct subsequences (i.e., patterns) indicates greater complexity.

8 $${S}_{{{{\rm{samp}}}}}=\log \frac{{\sum }_{i=1}^{N-m}{\sum }_{j=1,j\ne i}^{N-m}u\left(r-{d}_{{{\infty }}}\left({{{{\bf{X}}}}}_{m,i},{{{{\bf{X}}}}}_{m,j}\right)\right)}{{\sum }_{i=1}^{N-m}{\sum }_{j=1,j\ne i}^{N-m}u\left(r-{d}_{{{\infty }}}\left({{{{\bf{X}}}}}_{m+1,i},{{{{\bf{X}}}}}_{m+1,j}\right)\right)}$$

The upper threshold of *S*_samp_ is represented as log((*N* − *m* − 1)(*N* − *m*)/2). However, this maximum value can be regarded as an overestimated value rather than a theoretical maximum.

### Permutation entropy calculation

For the given data sequence, permutation entropy (*S*_perm_) can be used to quantify the complexity based on the probability of the probability distribution of ordinal patterns within the sequence^39^. Similar to the sample entropy, the input signal **X** is segmented into subsequences **X**_*m,i*_ of length *m*. For each **X**_*m,i*_, a permutation pattern π, which represents the order or rank of the values within the subsequence, is calculated. The probability of a specific permutation pattern π_*i*_, denoted as *p*(π_*i*_), is calculated by dividing the number of occurrences of π_*i*_ by the total number of subsequences. *S*_perm_ is defined based on Shannon’s information entropy as follows:

9 $${S}_{{{{\rm{perm}}}}}=-{\sum}_{i}p\left({\pi }_{i}\right)\log p\left({\pi }_{i}\right)$$

When all possible permutation patterns have the same probability *p*(π_*i*_), *S*_perm_ has its theoretical maximum. The total number of possible permutations is *m*! when there are no equal values. However, when equal values exist in the input sequence, the number of possible permutations can be calculated using the Bell number *f*(*n*), which represents the number of ways to group *n* elements^51^

10 $$f\left(n\right)={\sum}_{i=1}^{n}\left({n}\atop{i}\right) \, f \, \left(n-i\right),\, \qquad f \, \left(0\right)=1$$

where (*n*; *i*) represents the binomial coefficient.

By normalizing the *S*_perm_ by the theoretical maximum, the resulting value provides a measure of the absolute complexity of the system.

### Graph entropy calculation

The complexity of the point pattern can be quantified using the graph entropy, which, on the hexagonal lattice, has been reported to coincide with the thermodynamic entropy^41^. To apply the graph entropy to a given point array that does not exhibit a strong 6-fold symmetry, a Voronoi diagram was constructed to resemble a hexagonal lattice^42^. For each randomly chosen location within the system, a fixed number of neighboring nodes (*n*) are sampled to construct a sub-network. Here, sub-graphs with the same graph isomorphism were treated as equivalent. The probability of *n*-node isomorphism *i* occurring is then can be defined as follows:

11 $${p}_{i}\left(n\right)\approx \frac{{f}_{i}}{m}$$

where *f*_*i*_ is the frequency of the *i*-th isomorphism observed among graphs with *n*-nodes, and *m* is the total number of distinct isomorphisms.

As the number of neighboring nodes included in the sub-graph increases, the complexity of the probability distribution increases exponentially. Considering the linear increase in the entropy as a function of the number of neighboring nodes, the entropy density *S*_*d*_ (i.e., entropy per single particle) can be defined as the increment in entropy when one node is added to the sub-graph.

12 $$S\left(n\right)=-{k}_{B}\left({\sum}_{i}{p}_{i}\left(n\right) \, {\log }_{2} \, {p}_{i} \, \left(n\right)-\left(d-1\right)\log n\right)$$

13 $${S}_{d}={\lim}_{n\to {{\infty }}}S\left(n+1\right)-S\left(n\right)\approx \frac{\Delta S\left({n}^{*}\right)}{\Delta n}$$

where *d* denotes the dimension of the system, and n^*^ is the number of nodes where the linearity of the entropy persists.

For an ordered structure such as a hexagonal close-packed arrangement, a specific sub-graph isomorphism will dominate, while in a disordered system, all possible sub-graph isomorphisms will tend to appear with similar probabilities.

### Hamming distance calculation

The Hamming distance (HD) between two data strings is determined by dividing the number of differing bits by the length of the strings, and as this distance nears 0.5, the strings become more independent from each other.

## Supplementary information

Supplementary Information

Description of Additional Supplementary Files

Supplementary Video 1. PS colloid polycrystalline self-assembly process

Supplementary Video 2. Diffraction-pattern response generation by PUF label scanning

Transparent Peer Review file

## Source data

Source Data

## Data Availability

The data supporting the findings of this study are available within the Article and its Supplementary Information or from the authors. Source data are provided with this paper.
