# Supplementary material for: Dual-space visible light authentication toward high security physical unclonable function
Source: Nat Commun. 2026 Jul 23;17:8967. doi: 10.1038/s41467-026-75781-4 (PMC13503782; doi:10.1038/s41467-026-75781-4)
Supplement: Supplementary file 1 [file 41467_2026_75781_MOESM1_ESM.pdf]

Supplementary information

Dual-space visible light authentication toward high security physical unclonable function

Geon Gug Yang[1]†, Seong-Gyun Im[2]†, Taewoo Kang[2], Chan Woo Lee[1,3], Jonghwa Shin[1], Seok Joon Kwon[2,4]*, Sang Ouk Kim[1,3,5]*

[1] Department of Materials Science and Engineering, KAIST, Daejeon 34141, Republic of Korea.

[2] School of Chemical Engineering, Sungkyunkwan University (SKKU), Suwon 16419, Republic of Korea.

[3] KAIST Institute for Nanocentury, KAIST, Daejeon 34141, Republic of Korea.

[4] SKKU Institute of Energy Science & Technology (SIEST), SKKU, Suwon 16419, Republic of Korea.

[5] Materials Creation, Seoul, Republic of Korea.

* To whom correspondence should be addressed:

E-mail: sjoonkwon@skku.edu, sangouk@kaist.ac.kr

†These authors contributed equally to this work

Supplementary figures

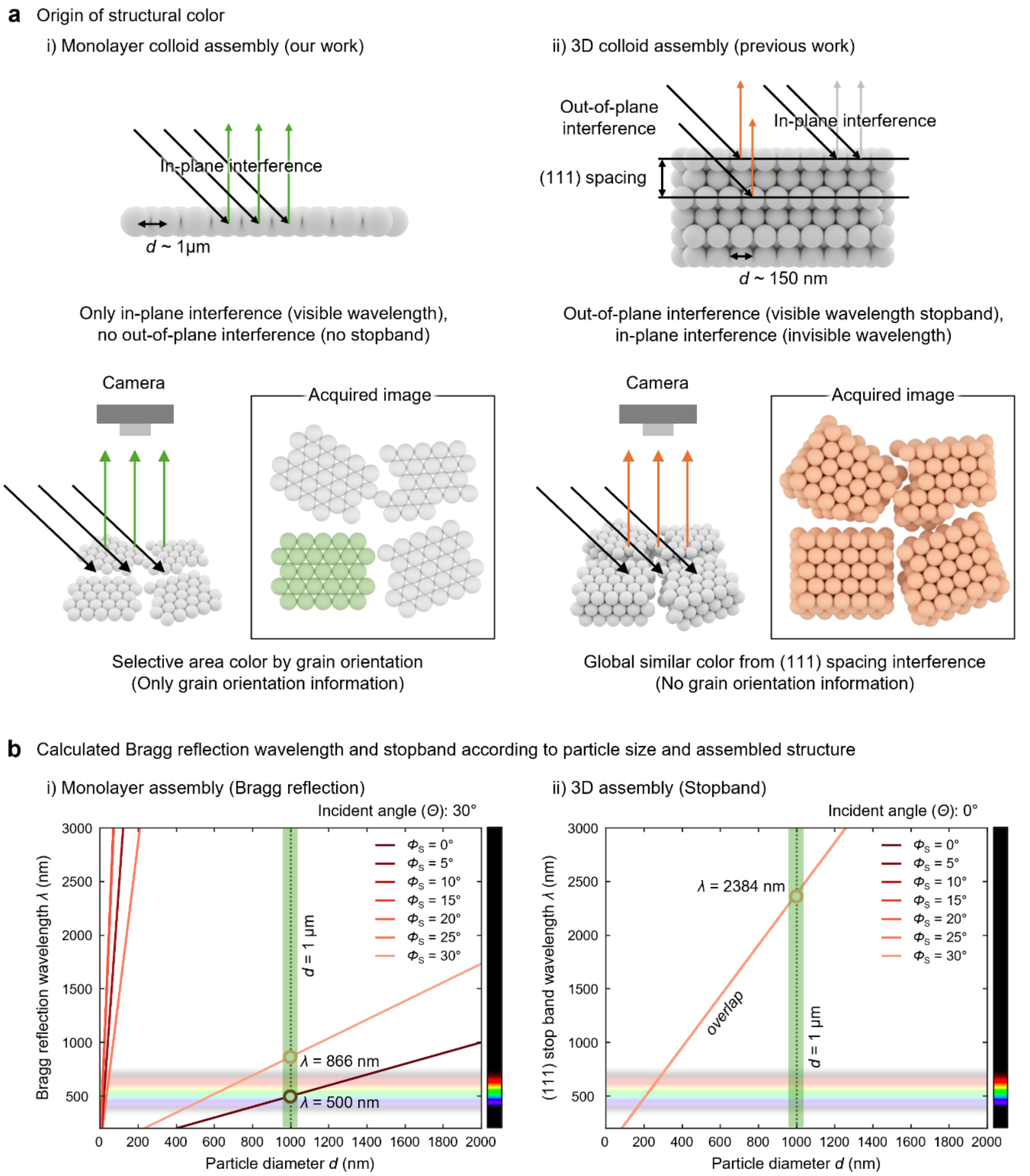

Supplementary Fig. 1 | **a**, Schematic illustration of origin of structural color in a monolayer colloid assembly (this work) and a 3D colloid assembly (previous work). **b,** Calculated (i) Bragg reflection wavelength and (ii) stopband as a function of particle size and assembled structure. Particle sizes that produce a stopband (out-of-plane constructive interference) do not generate Bragg reflection color (in-plane constructive interference), and vice versa. Structural color originating from the stopband remains unchanged with respect to the incident light angle modulation (*Φ*).

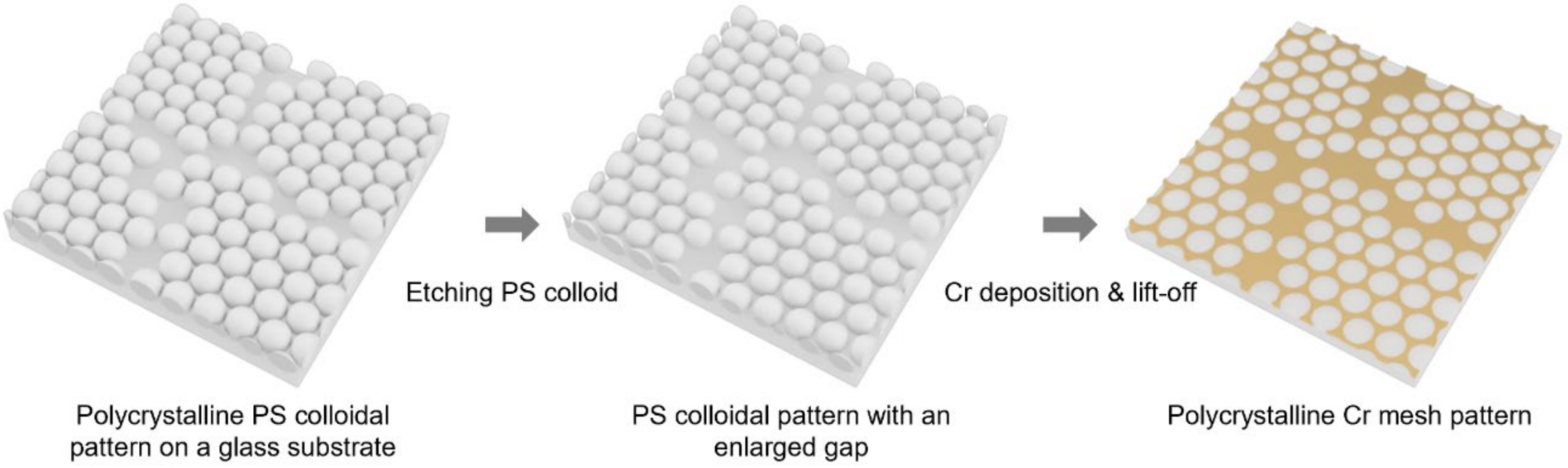

Supplementary Fig. 2 | Schematic illustration of the pattern transfer process from PS colloid pattern to Cr mesh pattern.

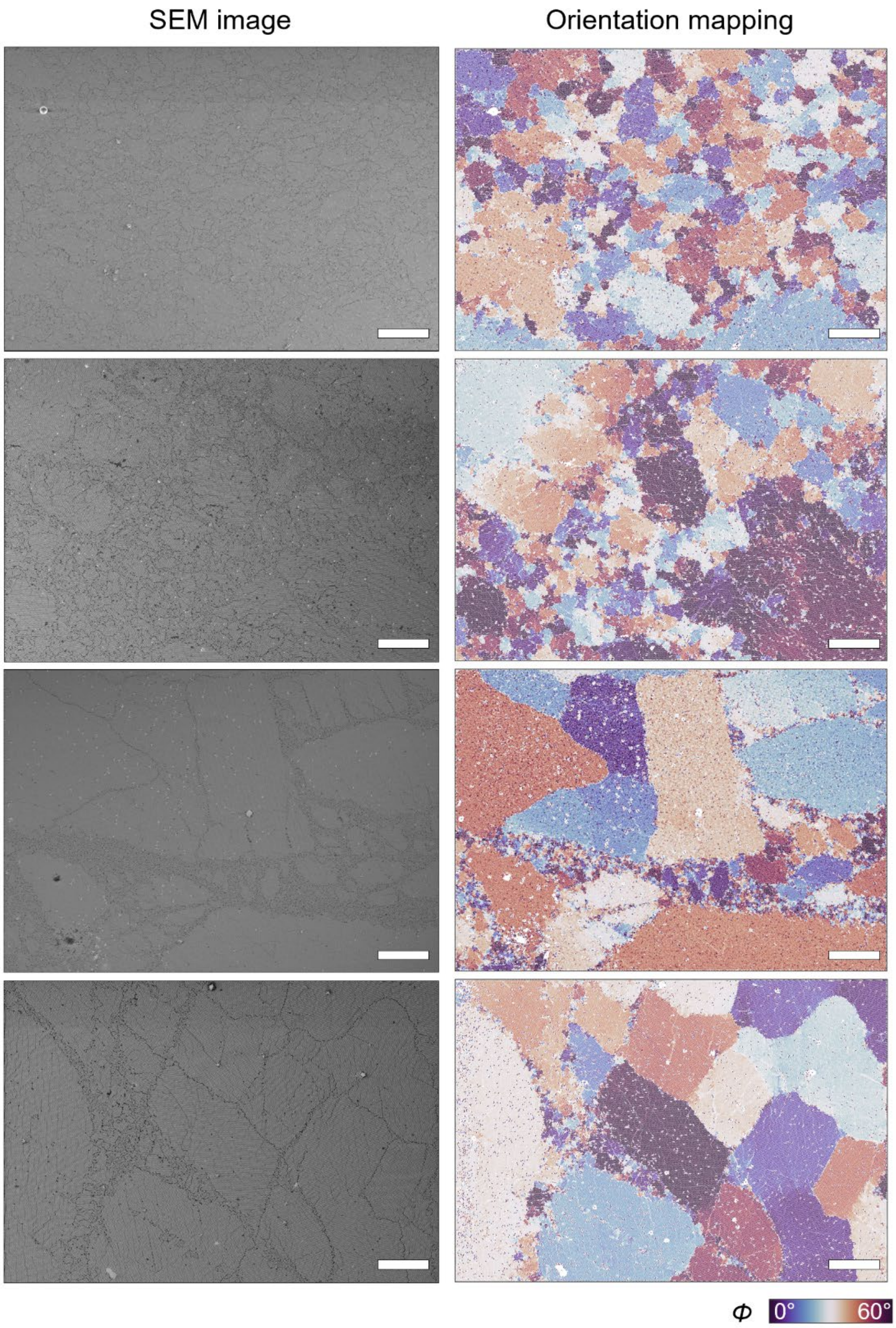

Supplementary Fig. 3 | SEM image and orientation mapping of a PUF label. (Left column) SEM image of a local area of the PUF label. Scale bars, 50 μm. (Right column) Corresponding orientation mapping image of the SEM image, where the color represents the orientations (Φ) of individual particles.

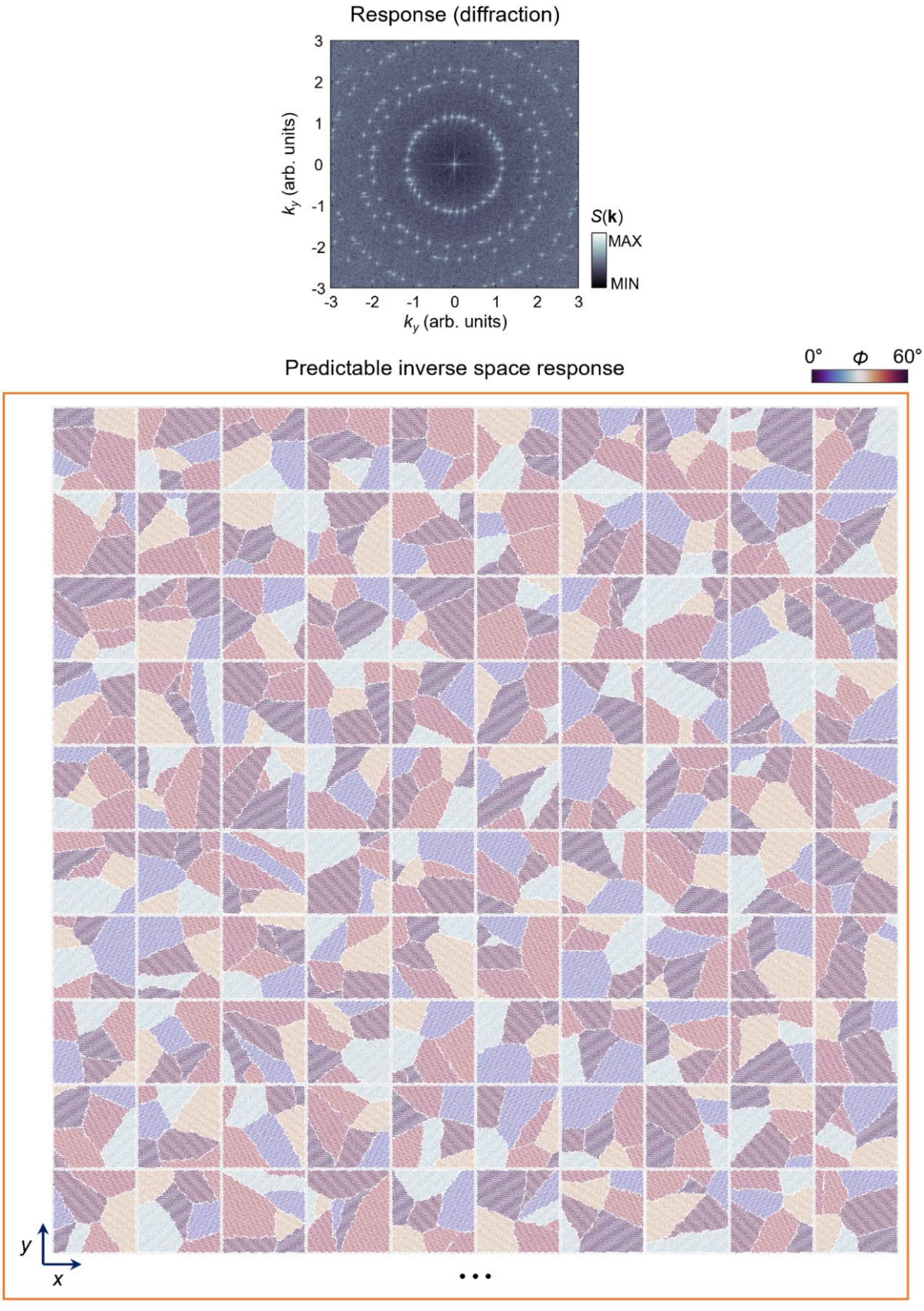

Supplementary Fig. 4 | Reconstructed real-space particle configurations from a specific diffraction pattern. Possible particle arrangements in real-space which can be reconstructed from a specific diffraction pattern obtained from the simulation (top panel). The color in the lower panel represents the orientations (Φ) of individual particles. There exists an infinite number of distinct particle configurations generating the same diffraction pattern, although representative 100 configurations are shown.

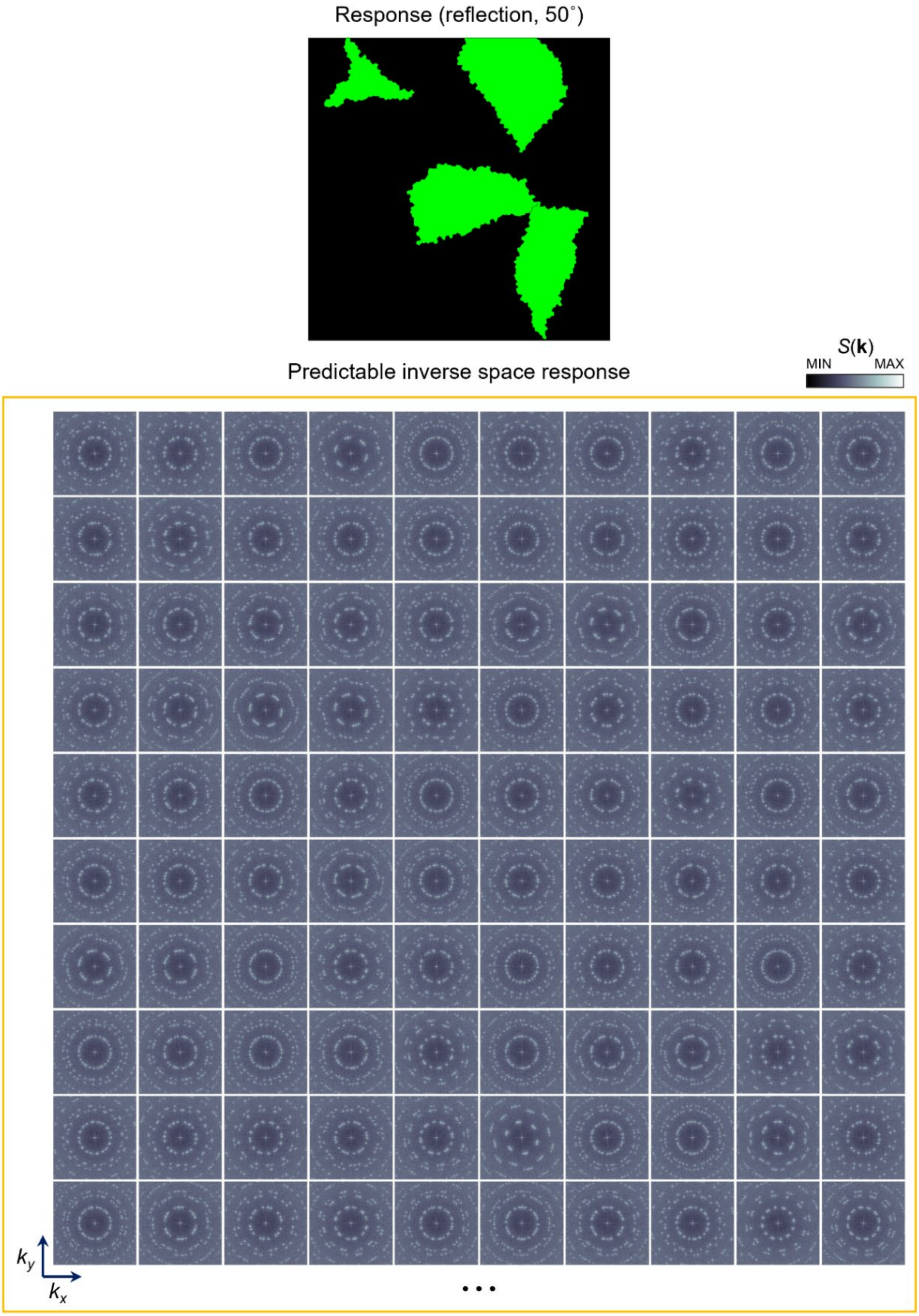

Supplementary Fig. 5 | Reconstructed reciprocal space response from a specific Bragg reflection pattern. Possible candidates of diffraction patterns, which can be reconstructed from a specific Bragg reflection response observed at an angle of $\theta = 30^\circ$, $\Phi = 50^\circ$ (top panel). There exists an infinite number of distinct diffraction patterns generating the same Bragg reflection pattern, although representative 100 reciprocal space responses are shown.

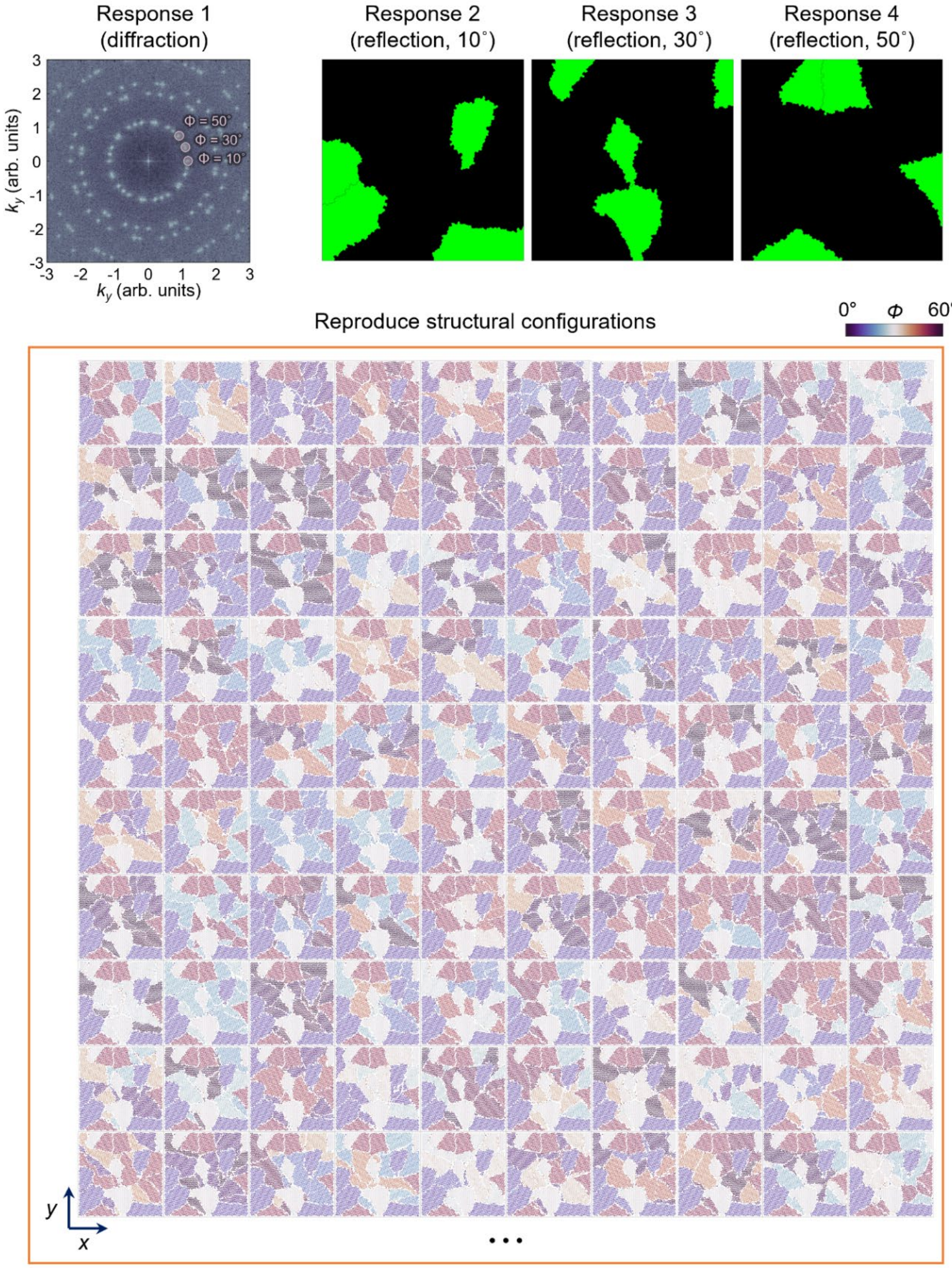

Supplementary Fig. 6 | Computational analysis of structural reconstruction resistance. Optical responses from diffraction and Bragg reflection modes at different angles (top panel) and representative structural configurations capable of producing identical responses (bottom panel). The color in the structural configurations represents the orientations (Φ) of individual particles. There exists an infinite number of distinct particle arrangements generating the same optical responses, although representative configurations are shown, confirming reconstruction impossibility from partial optical data.

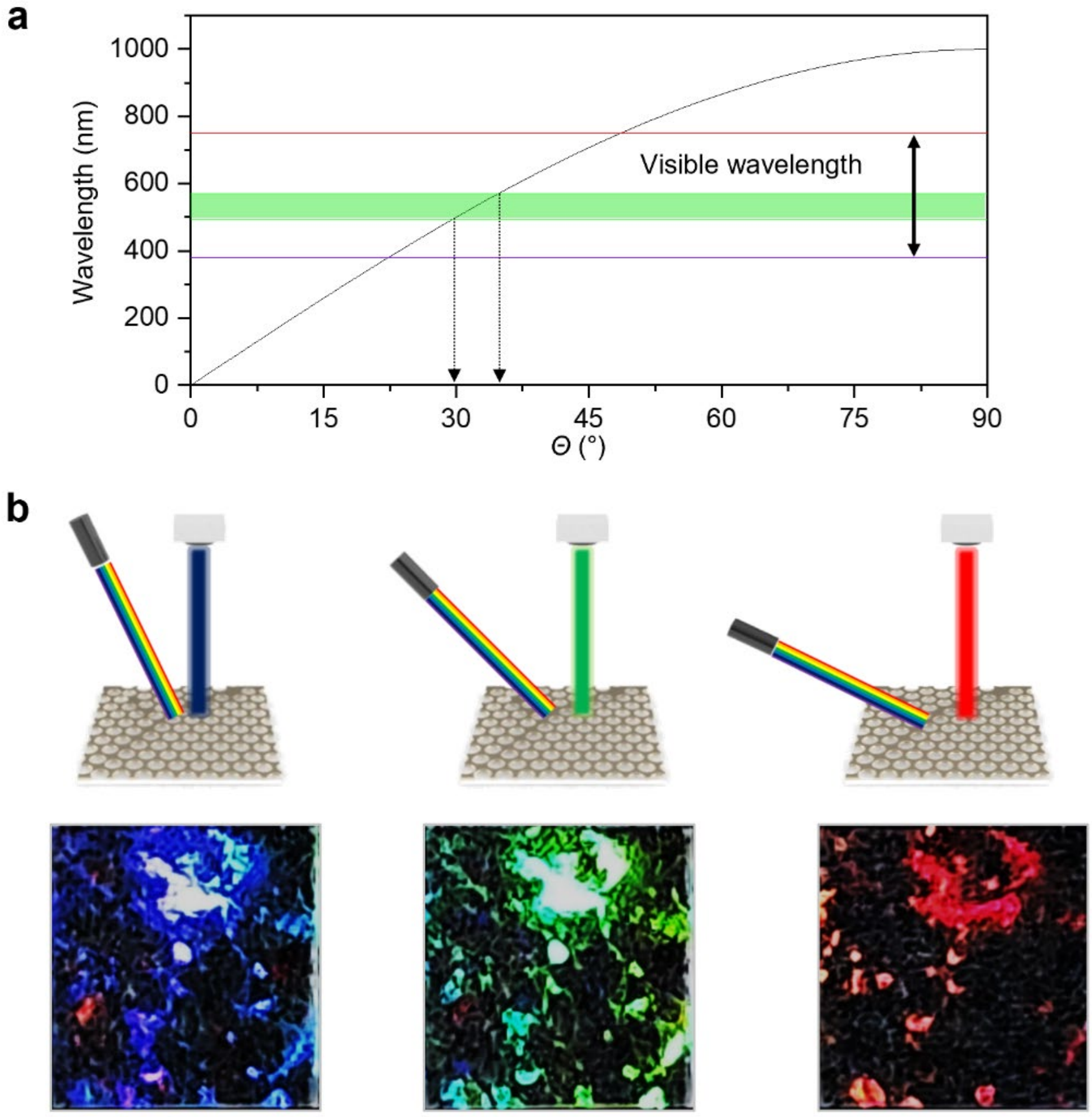

Supplementary Fig. 7 | Calculated constructive interference wavelength as a function of light polar angle (θ) and photograph image of the PUF label at a specific light polar angle (θ). a, Calculation results of the constructive interference wavelength as a function of the light polar angle (θ), assuming that the crystal orientation (Φ) aligns with the light azimuthal angle (Φ_s). **b,** Bragg reflection pattern image of the PUF label at a specific light polar angle (θ).

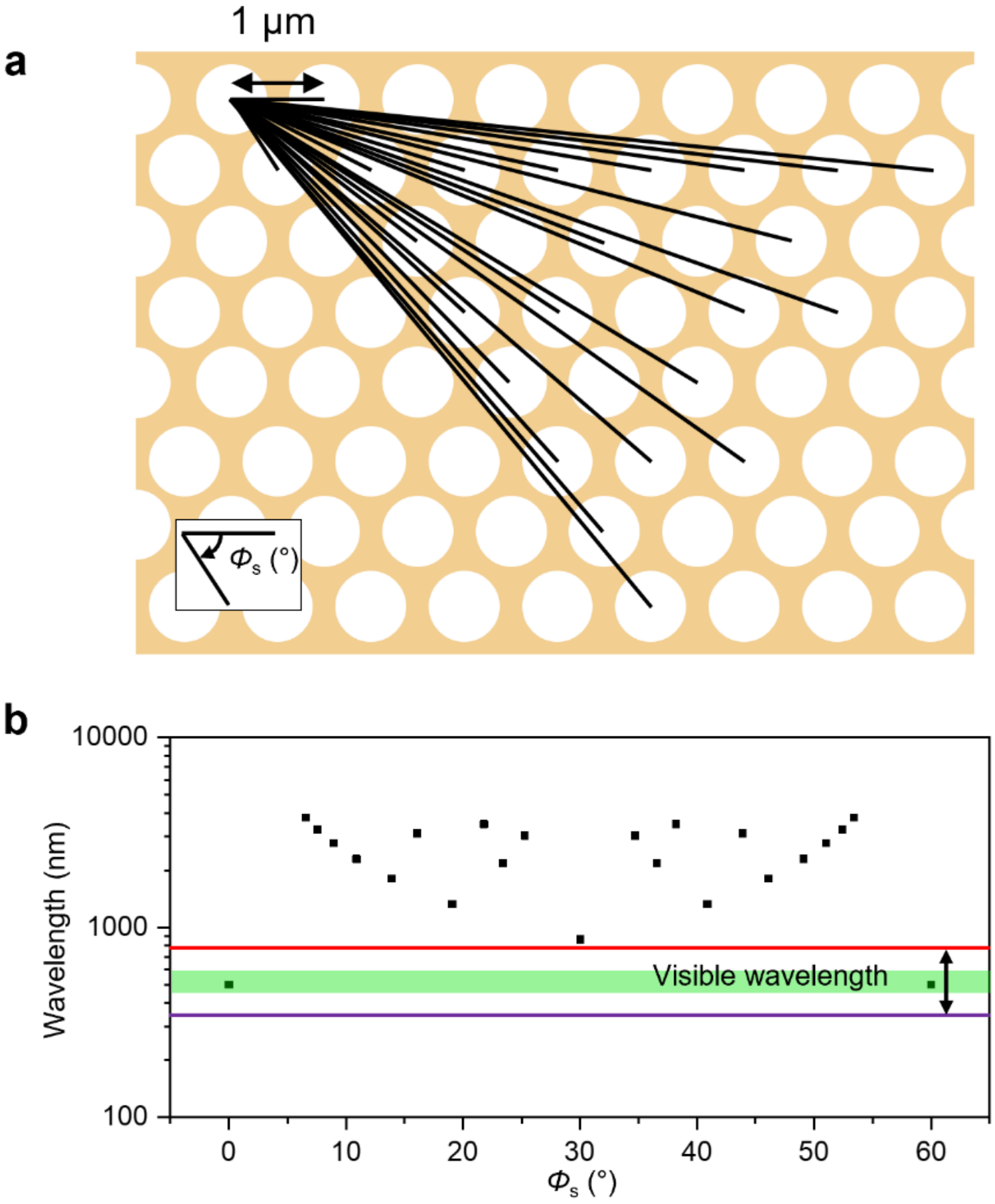

Supplementary Fig. 8 | Calculated constructive interference wavelength as a function of crystal orientation (Φ) at a fixed polar angle (θ). a, Schematic illustration of the PUF label and the possible constructive interference period. **b,** Calculation results of the constructive interference wavelength based on the crystal orientation (Φ). The wavelength deviates significantly from the visible light range except when the light azimuthal angle (Φ_s) and crystal angle (Φ) are precisely aligned.

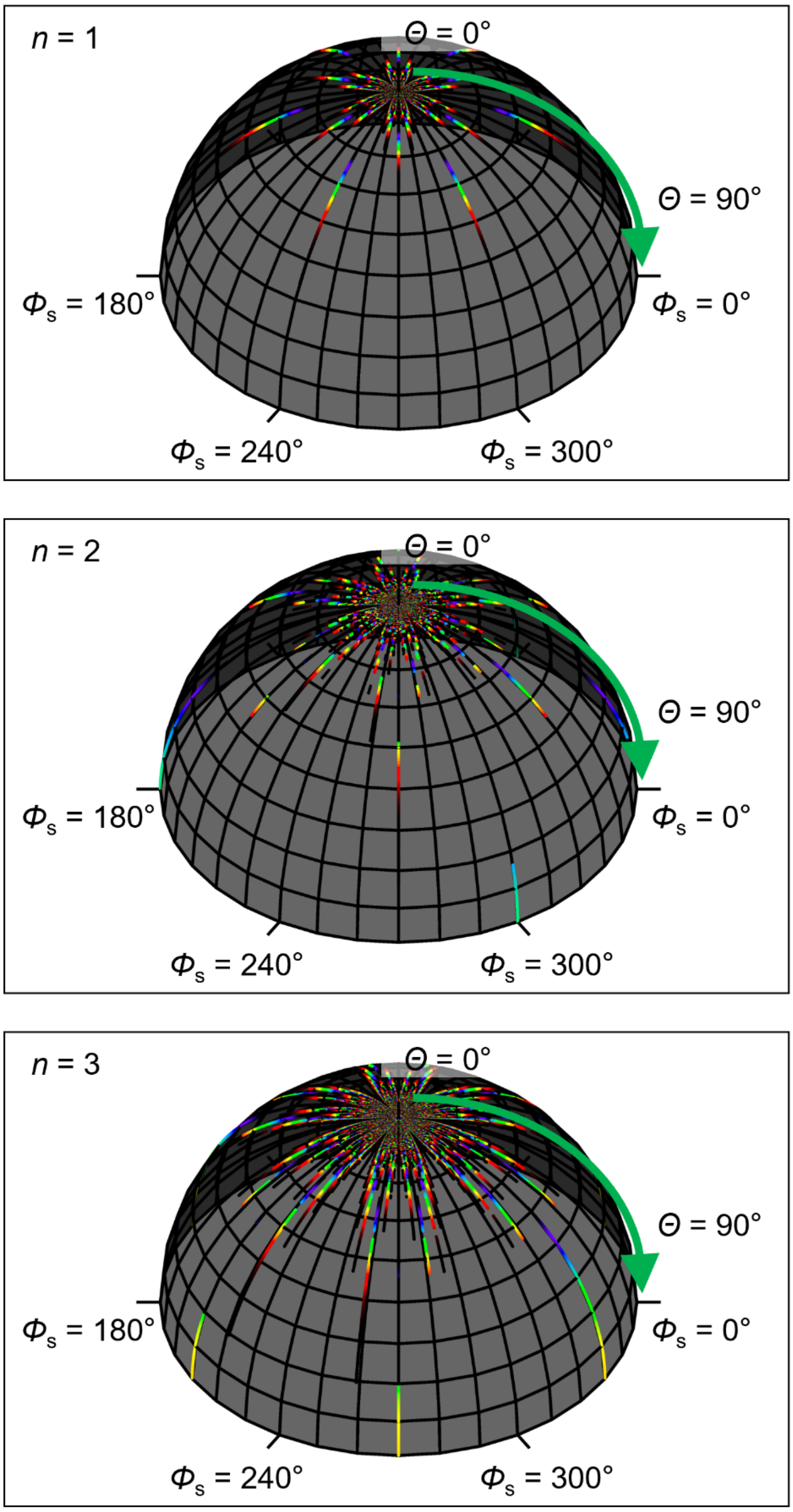

Supplementary Fig. 9 | Simulation of local interference colors in HCP structures under varying light incidence angles. Simulated local interference colors with the incident light angle of (θ, Φ) on the hexagonal close-packed (HCP) structure. Only the visible region was considered. The results were presented for the first three modes: n = 1 (top), n = 2 (middle), and n = 3 (bottom). Each hemisphere represents the angular distribution of the interference colors, corresponding to the specified mode number.

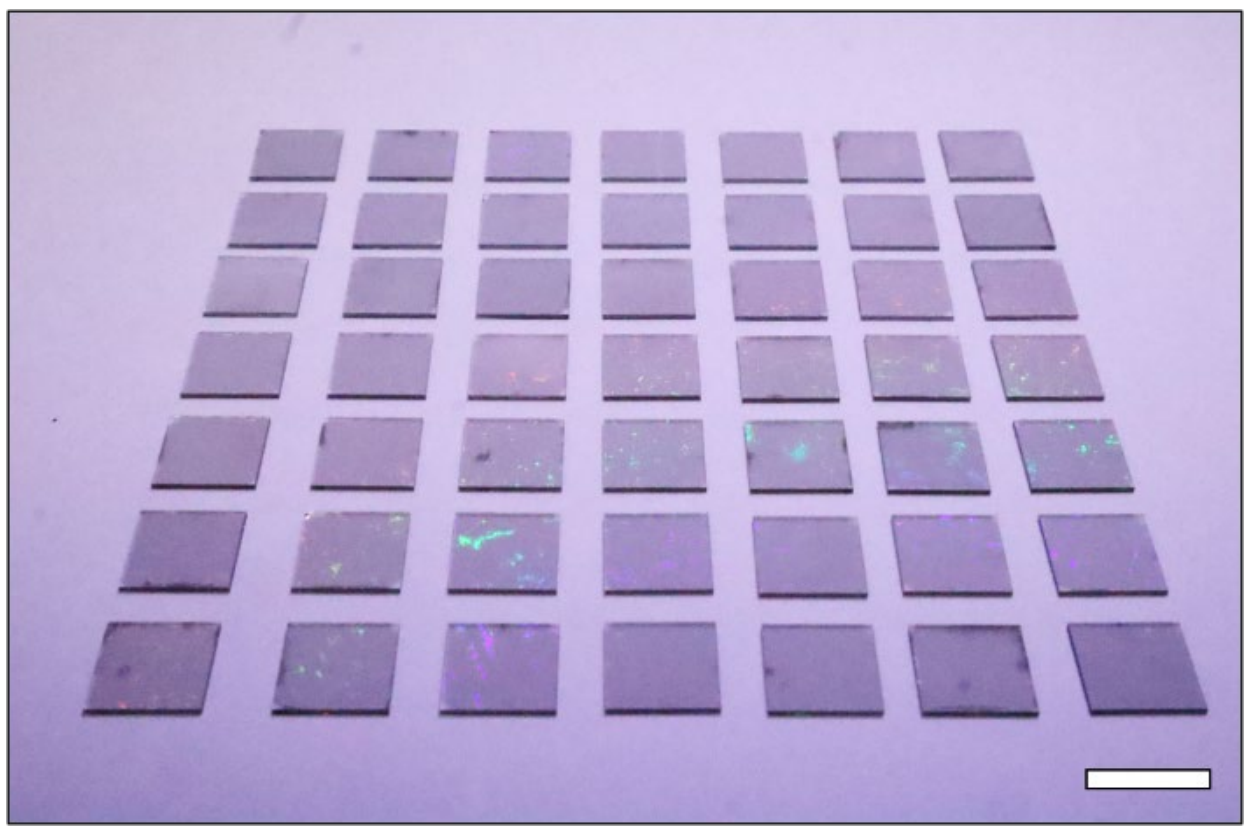

Supplementary Fig. 10 | Mass production of PUF label. Photograph image of 49 PUF labels with 1cm × 1cm size. Scale bar, 1 cm.

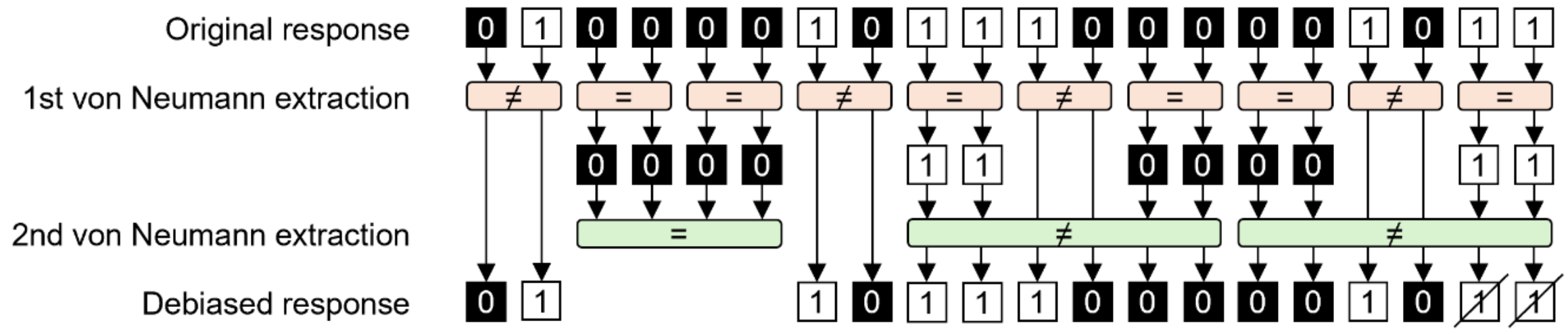

Supplementary Fig. 11 | Schematic of the multi-pass tuple-output von Neumann (MO-TO-VN) debiasing. The first von Neumann extractor checks the original response to see if the consecutive bit pairs are identical. If both bits are opposed, they are retained as debiased bits. The pairs with identical bits are grouped into quadruplets with four bits, and the extractor checks the first half of a quadruplet to the second half. If the first half and the second half of the quadruplet are the same, they are discarded, while if they are different, all four bits in the quadruplet are retained. Note that the last bit pair is not retained.

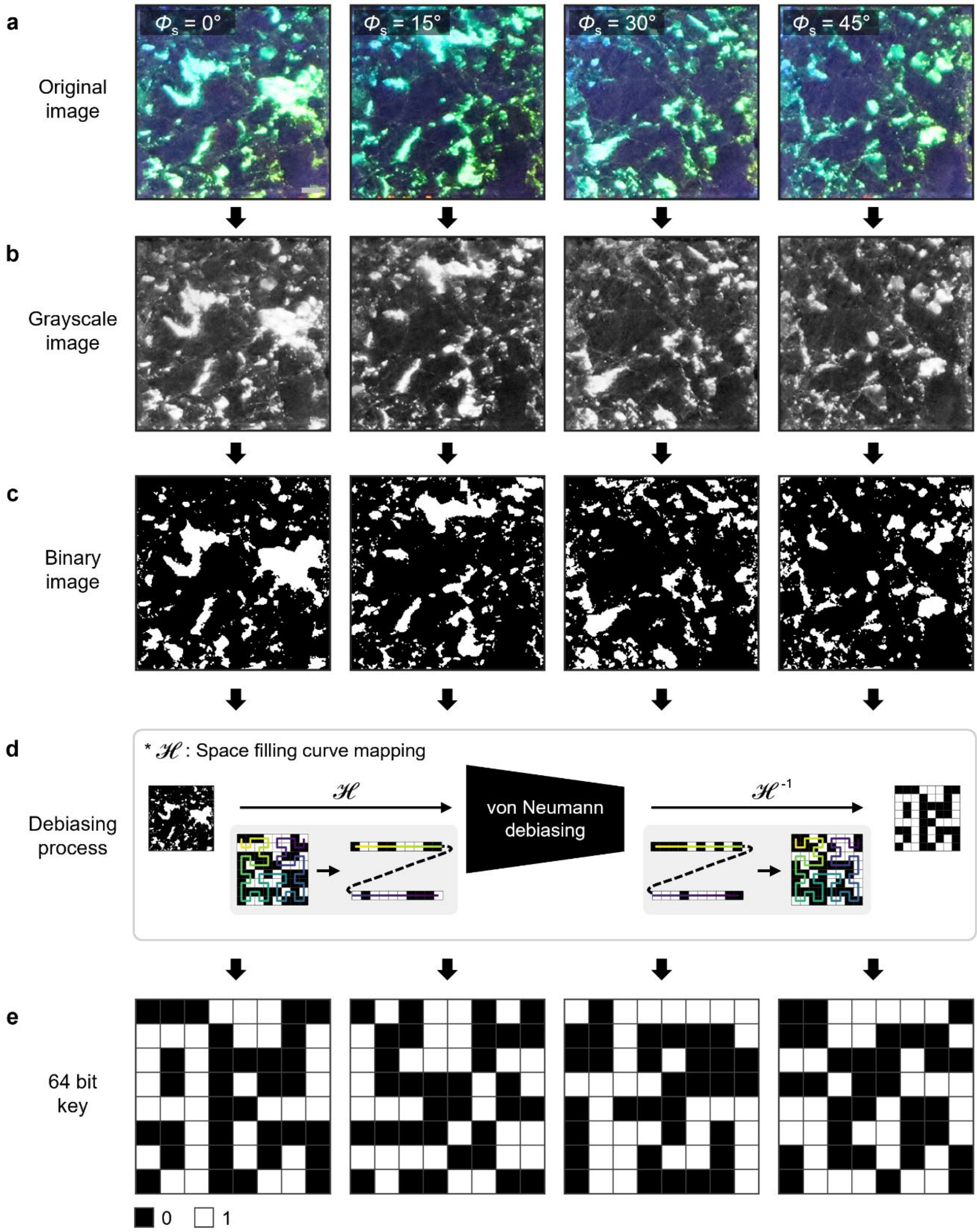

Supplementary Fig. 12 | Bragg reflection pattern-based key generation process. Schematic of the Bragg reflection pattern-based key generation process. **a,** The original optical images at varying azimuthal angles Φ_s (0°, 15°, 30°, and 45°) and constant polar angle θ were processed into **b,** grayscale images and **c,** binary images with further image processing. The 2D binary image matrix is transformed into a 1D vector via space-filling curve (i.e., Hilbert curve) mapping $\mathscr{H}$, followed by von Neumann debiasing. **d,** The first 64-bit of the flattened image is reconstructed back to the debiased 2D binary image by an inverse space-filling curve mapping $\mathscr{H}^1$, **e,** which leads to the final 64-bit key.

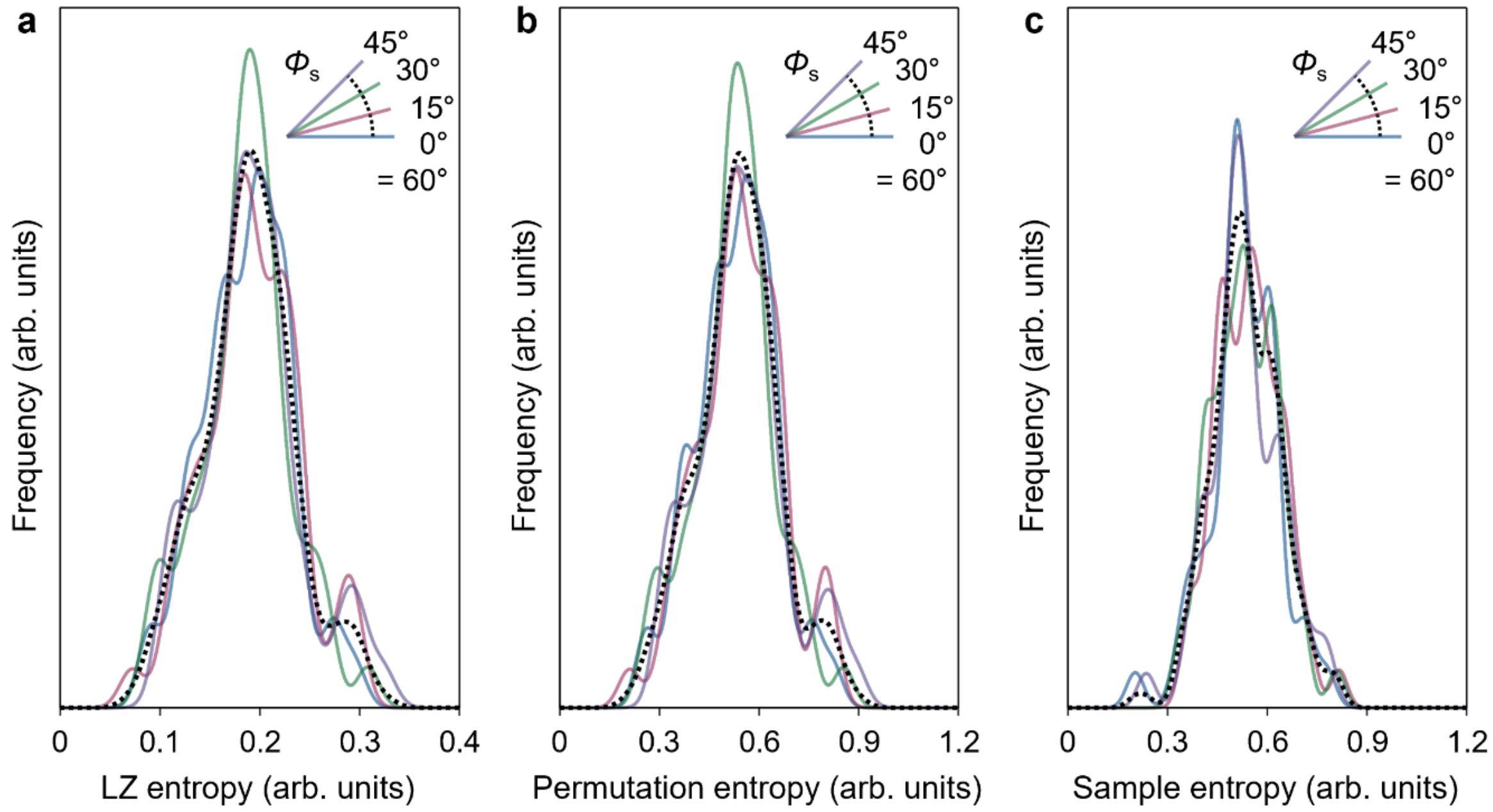

Supplementary Fig. 13 | Probability density distribution of entropy of Bragg reflection keys. Probability density distribution of entropy of Bragg reflection keys generated under the incident white light with four azimuthal angles Φ_s (0°, 15°, 30°, and 45°) and a fixed polar angle θ = 30°. Due to the 6-fold symmetry represented in the system, Φ_s = 0° and Φ_s = 60° produce equivalent results. The solid curves correspond to different azimuthal angles: red for 0°, orange for 15°, green for 30°, and blue for 45°. The dotted black line represents the average entropy distribution across four azimuthal angles. **a,** Lempel-Ziv (LZ) entropy, **b,** permutation entropy, and **c,** sample entropy.

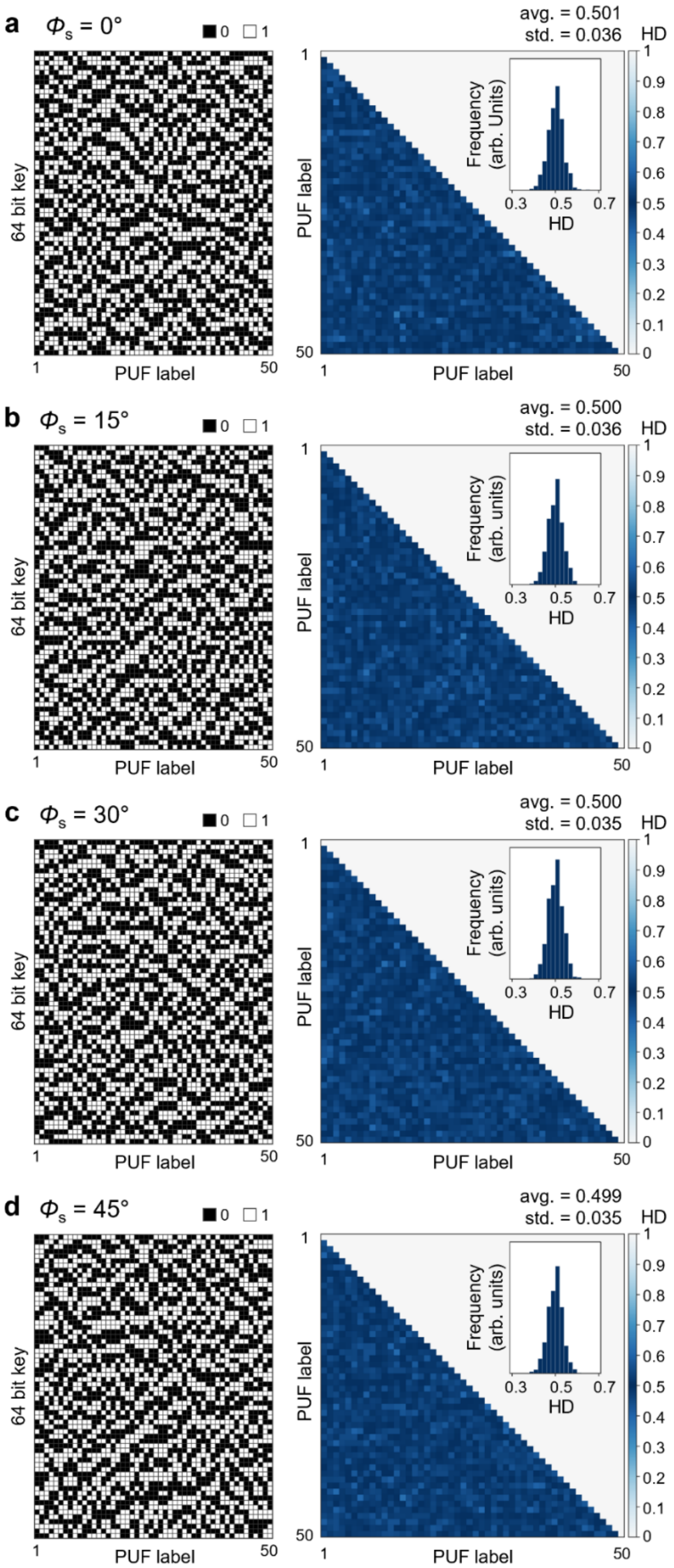

Supplementary Fig. 14 | Hamming distance (HD) of Bragg reflection keys at varying azimuthal angles. (Left column) 64-bit keys, obtained from 50 PUF labels, generated from Bragg reflection patterns under specific azimuthal angle Φ_s (**a,** 0˚, **b,** 15˚, **c,** 30˚, and **d,** 45˚) and a constant polar angle ($\theta = 30˚$). (Right column) HD analysis of 50 Bragg reflection pattern-based keys under the specific incident light angle of Φ_s. The histogram in the inset represents the distribution of HD with the average and standard deviation.

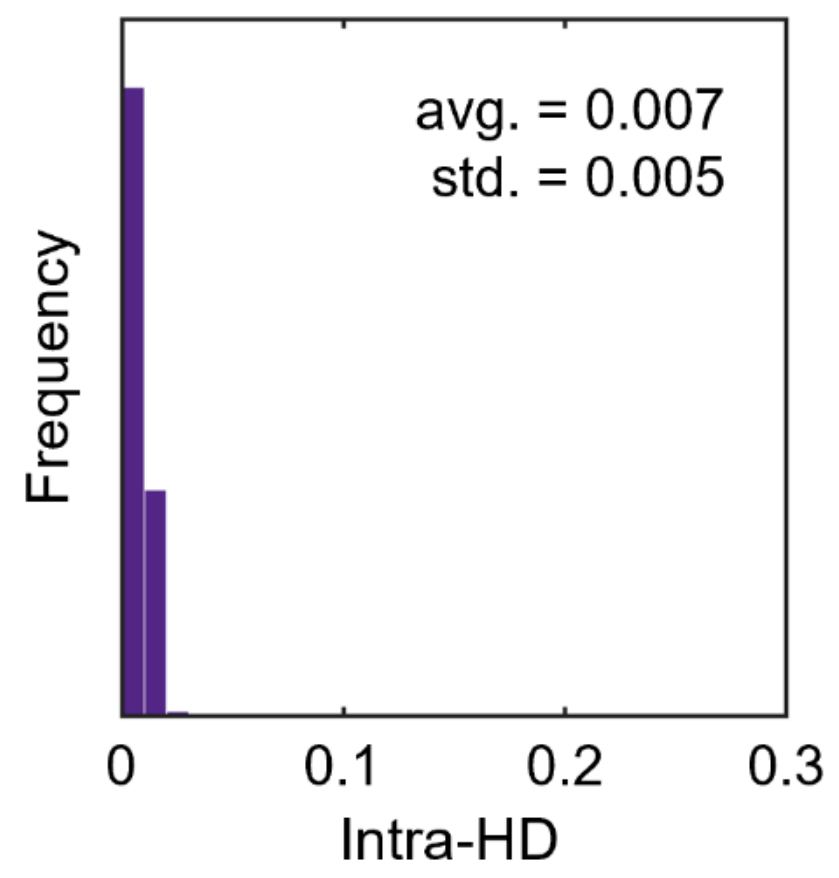

Supplementary Fig. 15 | Intra-Hamming distance (HD) distribution for r-space authentication.

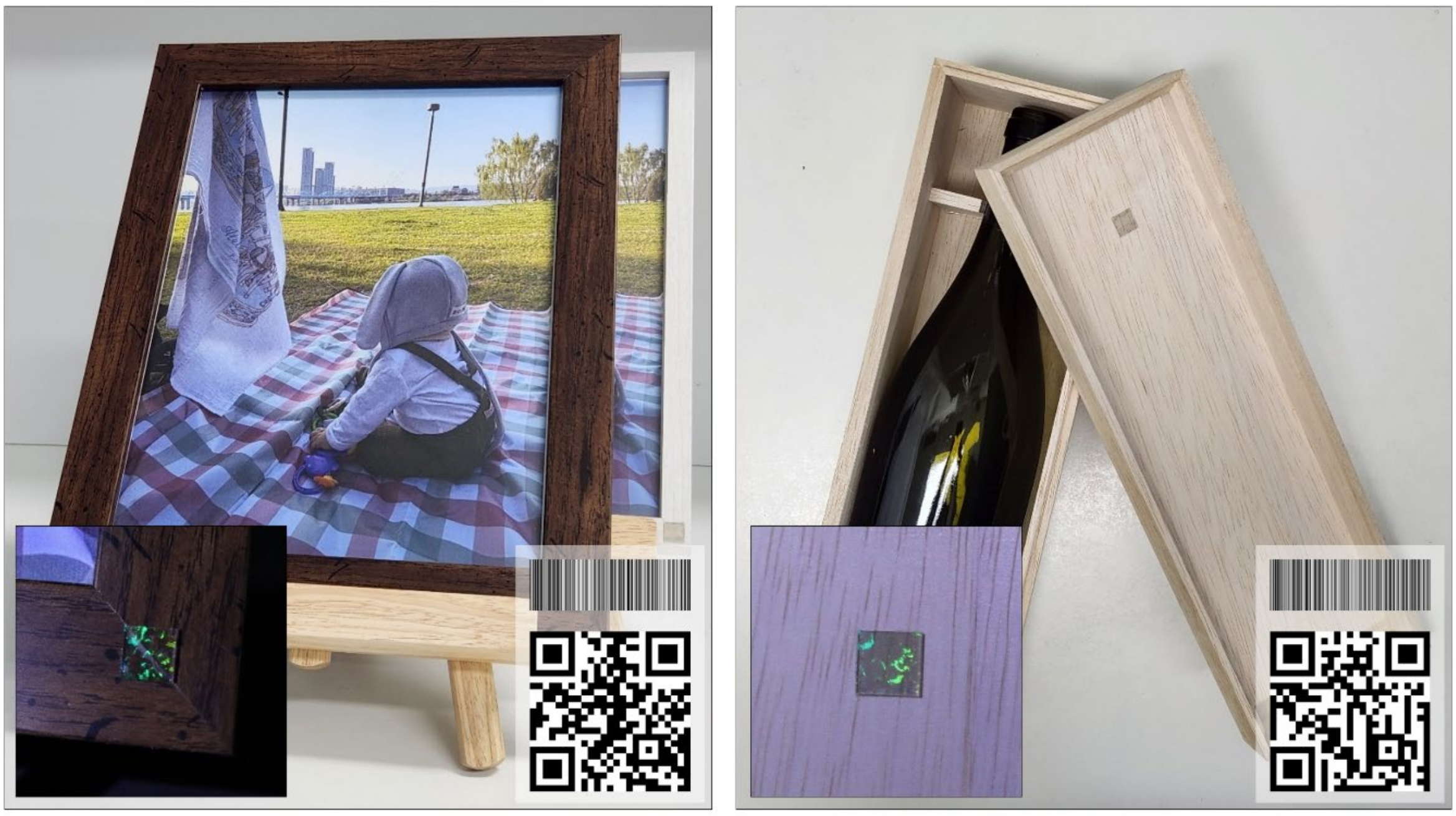

Supplementary Fig. 16 | Practical demonstration of PUF label usage based on Bragg reflection pattern.

Test image (Supplementary Fig. 17a) – Background image (Supplementary Fig. 17b)
= Diffraction image (Supplementary Fig. 17c)

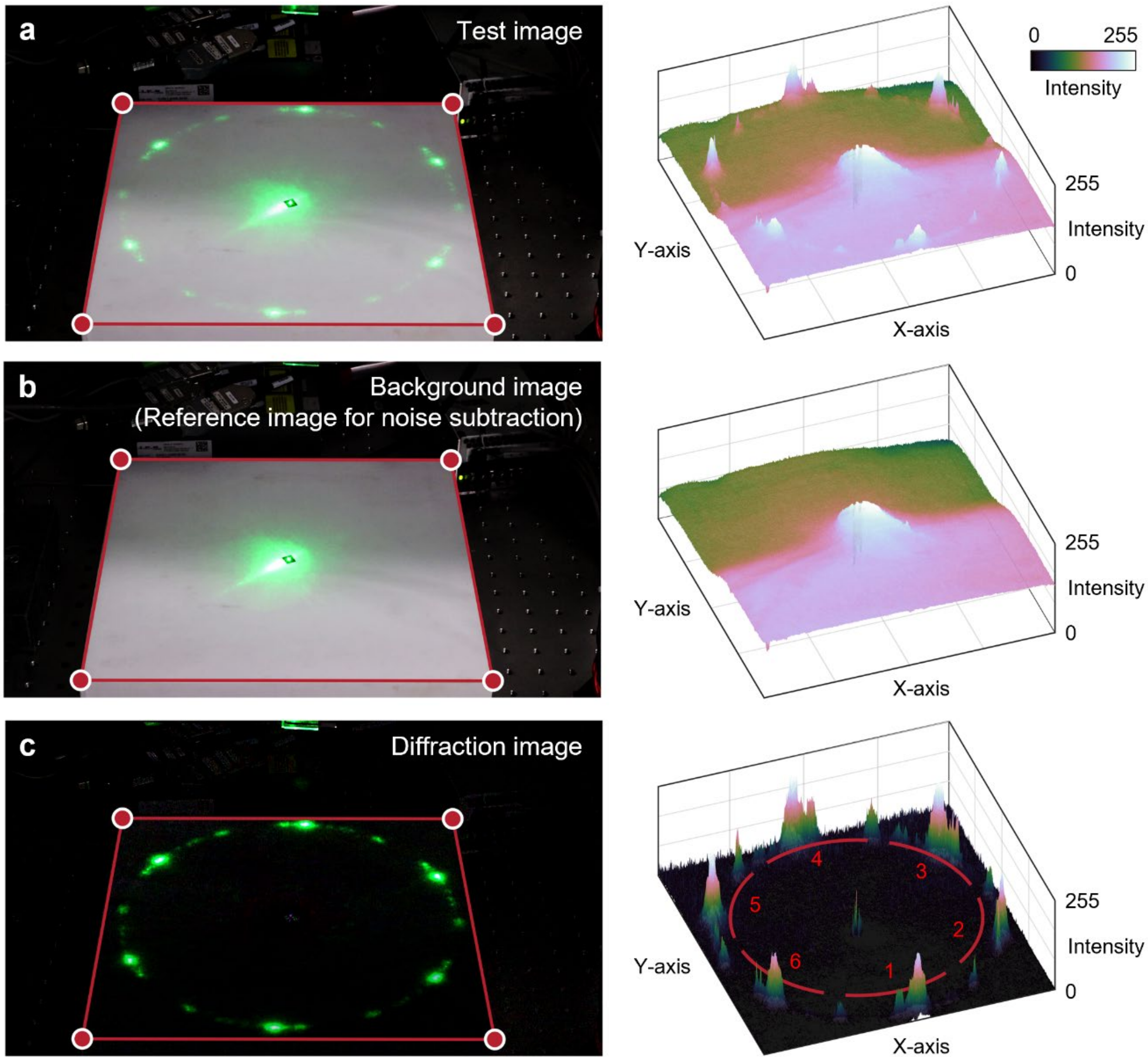

Supplementary Fig. 17 | Image processing protocol for diffraction pattern authentication. a, Raw diffraction pattern image (left) and corresponding 3D intensity surface plot (right). **b,** Background reference image (left) and corresponding 3D intensity surface plot (right). **c,** Enhanced pattern image (left) and corresponding 3D intensity surface plot (right), where *k* is an enhancement factor.

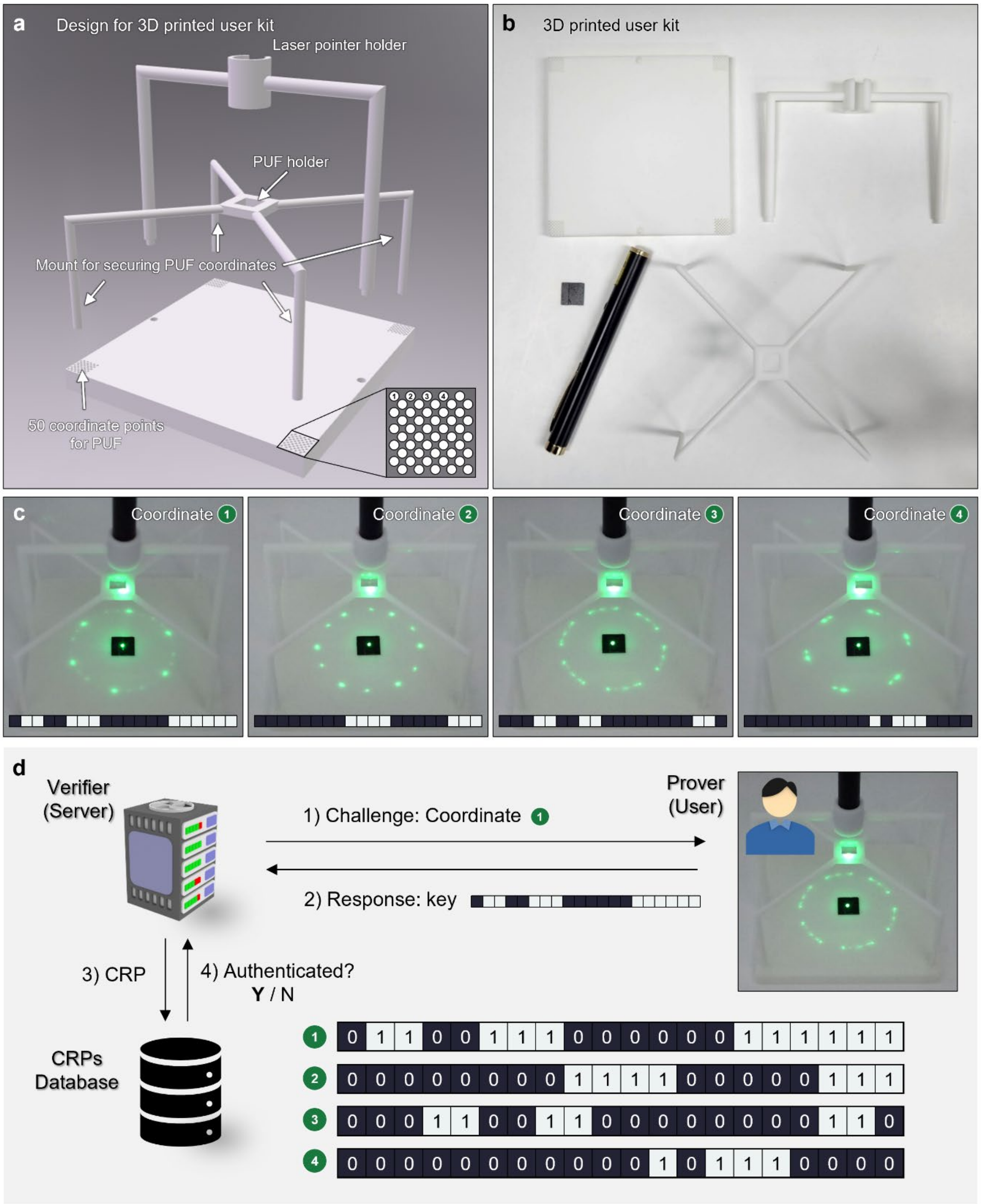

Supplementary Fig. 18 | 3D printed user kit for diffraction pattern based authentication. **a,** Design image of the 3D printed user kit. **b,** Photograph image of the 3D printed user kit. **c,** Diffraction pattern of the PUF label at several coordinates. **d,** Authentication protocol using the 3D printed user kit. 1) The Verifier (Server) sends a coordinate challenge to the Prover (User), 2) who uses the kit to capture the diffraction pattern at that coordinate as a response key. 3) The Verifier performs CRP lookup in its database, comparing the received response against pre-registered diffraction patterns for each coordinate. 4) Upon finding a matching CRP, the system authenticates the user.

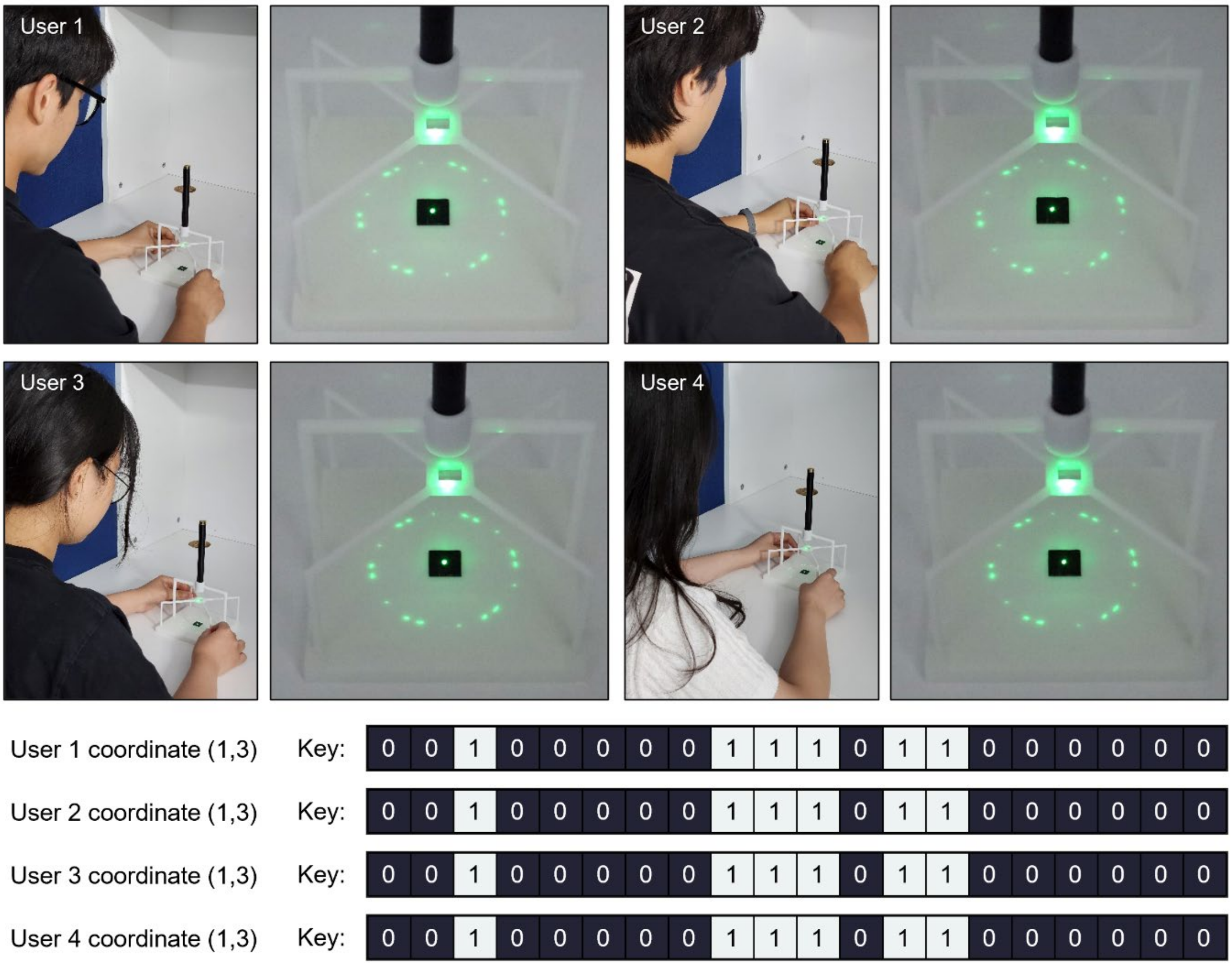

Supplementary Fig. 19 | Verification of user kit reproducibility depending on the user

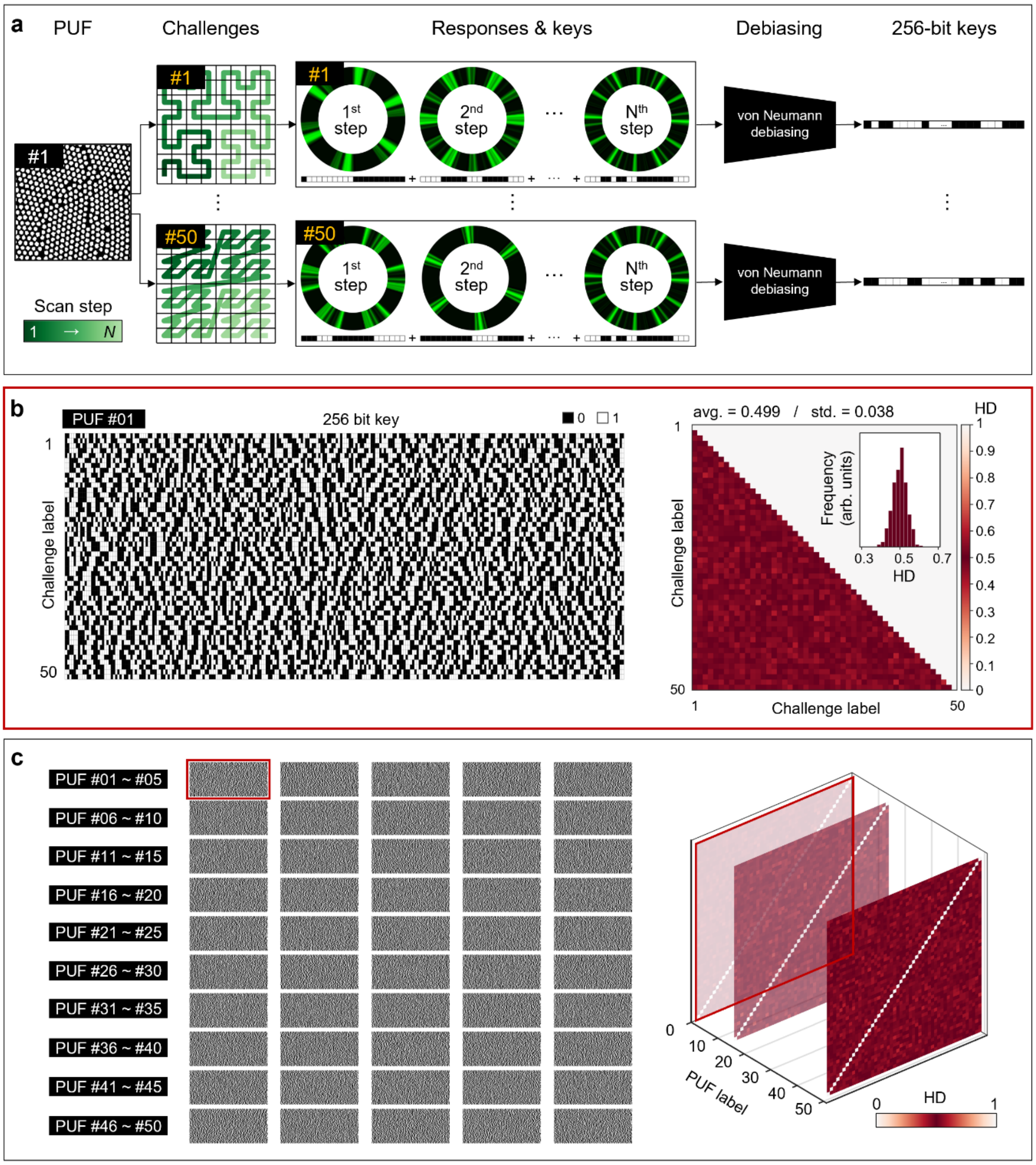

Supplementary Fig. 20 | Diffraction key generation process and HD analysis for 50 PUFs across multiple challenges. a, Schematic diagram of the 256-bit key generation process from a single PUF with 50 different challenges. The challenge is a permutation of N spots at which the laser is illuminated, generating distinct diffraction responses converted into binary keys. The binary keys are concatenated and debiased using von Neumann debiasing, with only the first 256 bits used as the final key. **b,** 256-bit keys generated from PUF #01 with 50 different challenges (left) and their HD distribution (right). The inset shows the HD distribution of the keys. **c,** 256-bit key sets obtained from 50 challenges for 50 PUF labels (left). The HD distribution of representative PUF labels (PUF #01, #15, and #50) is shown on the right. The detailed information on PUF #01 (red box) is shown in **b**.

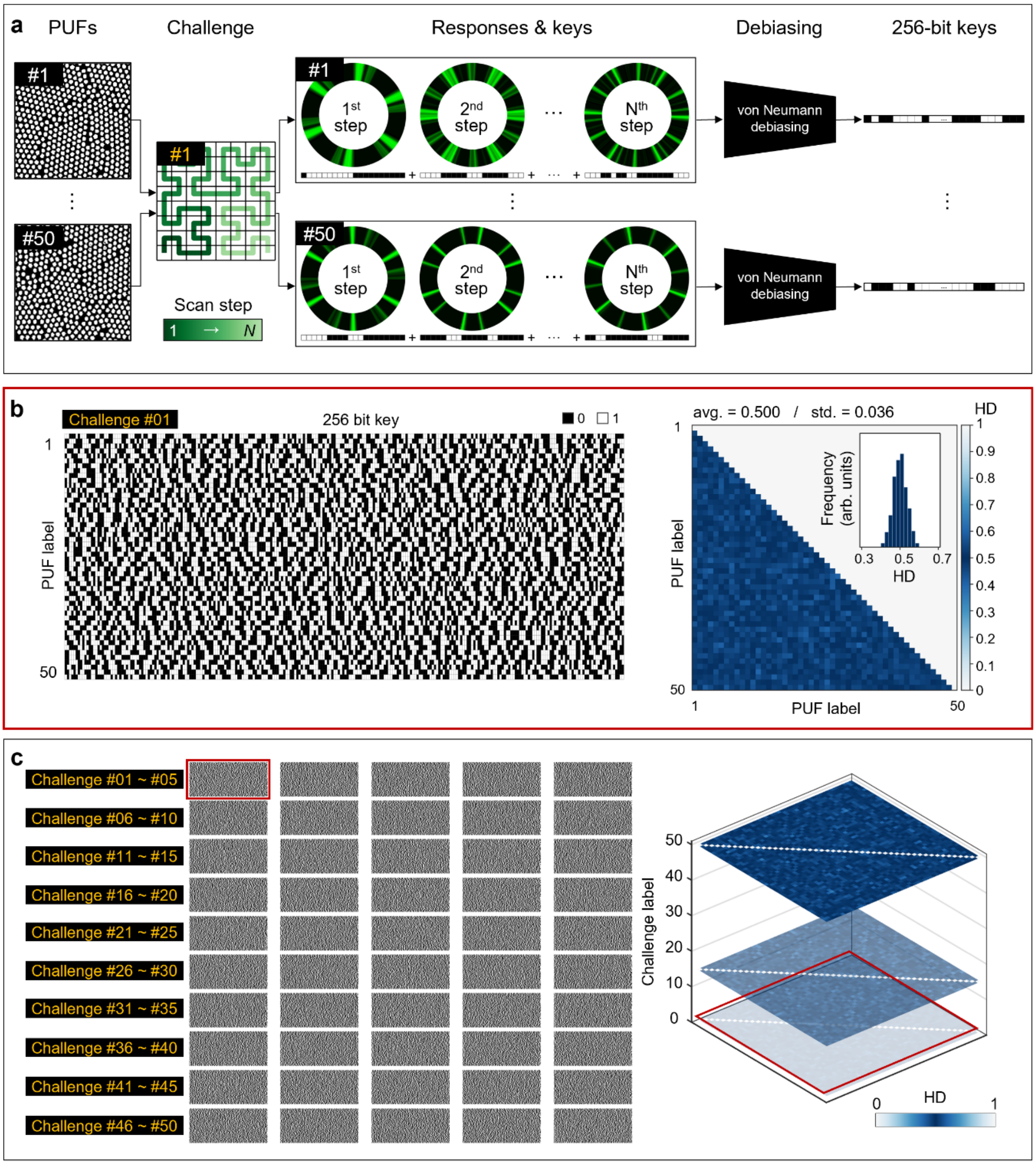

Supplementary Fig. 21 | Diffraction key generation process and HD analysis for 50 challenges across multiple PUFs. a, Schematic diagram of the 256-bit key generation process from 50 different PUF labels with a single challenge. The challenge is a permutation of N spots at which the laser is illuminated, generating distinct diffraction responses converted into binary keys. The binary keys are concatenated and debiased using von Neumann debiasing, with only the first 256 bits used as the final key. **b,** 256-bit keys generated from PUF #01 to #50 with a single challenge (left) and their HD distribution (right). The inset shows the HD distribution of the keys. **c,** 256-bit key sets obtained from 50 PUF labels for 50 challenges (left). The HD distribution of representative challenge labels (Challenge #01, #15, and #50) is shown on the right. The detailed information on Challenge #01 (red box) is shown in **b**.

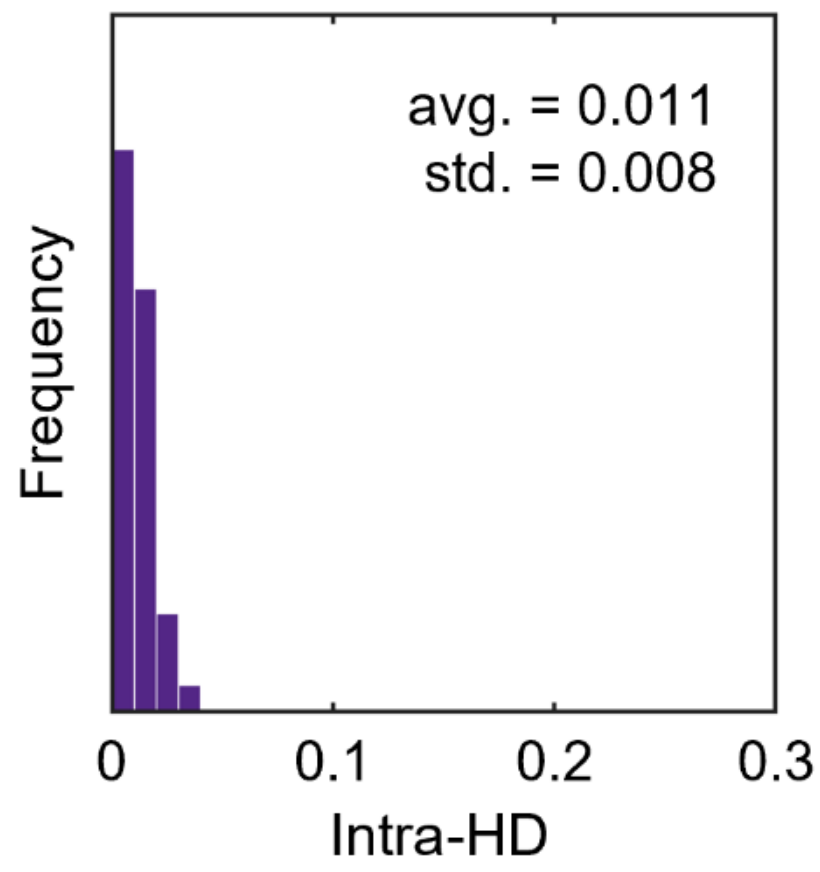

Supplementary Fig. 22 | Intra-Hamming distance (HD) distribution for k-space authentication.

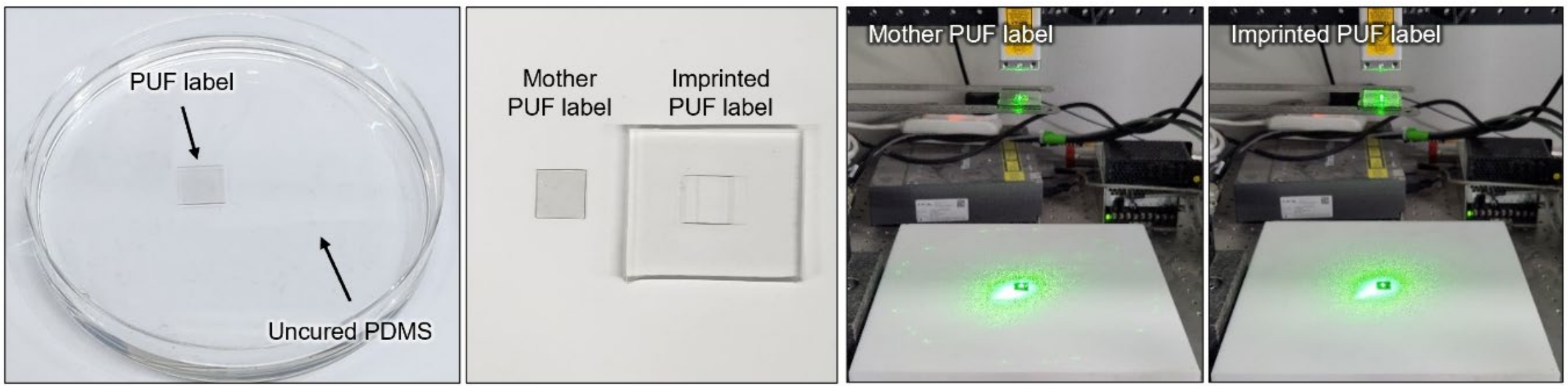

Supplementary Fig. 23｜PDMS imprinting based pattern replication test.

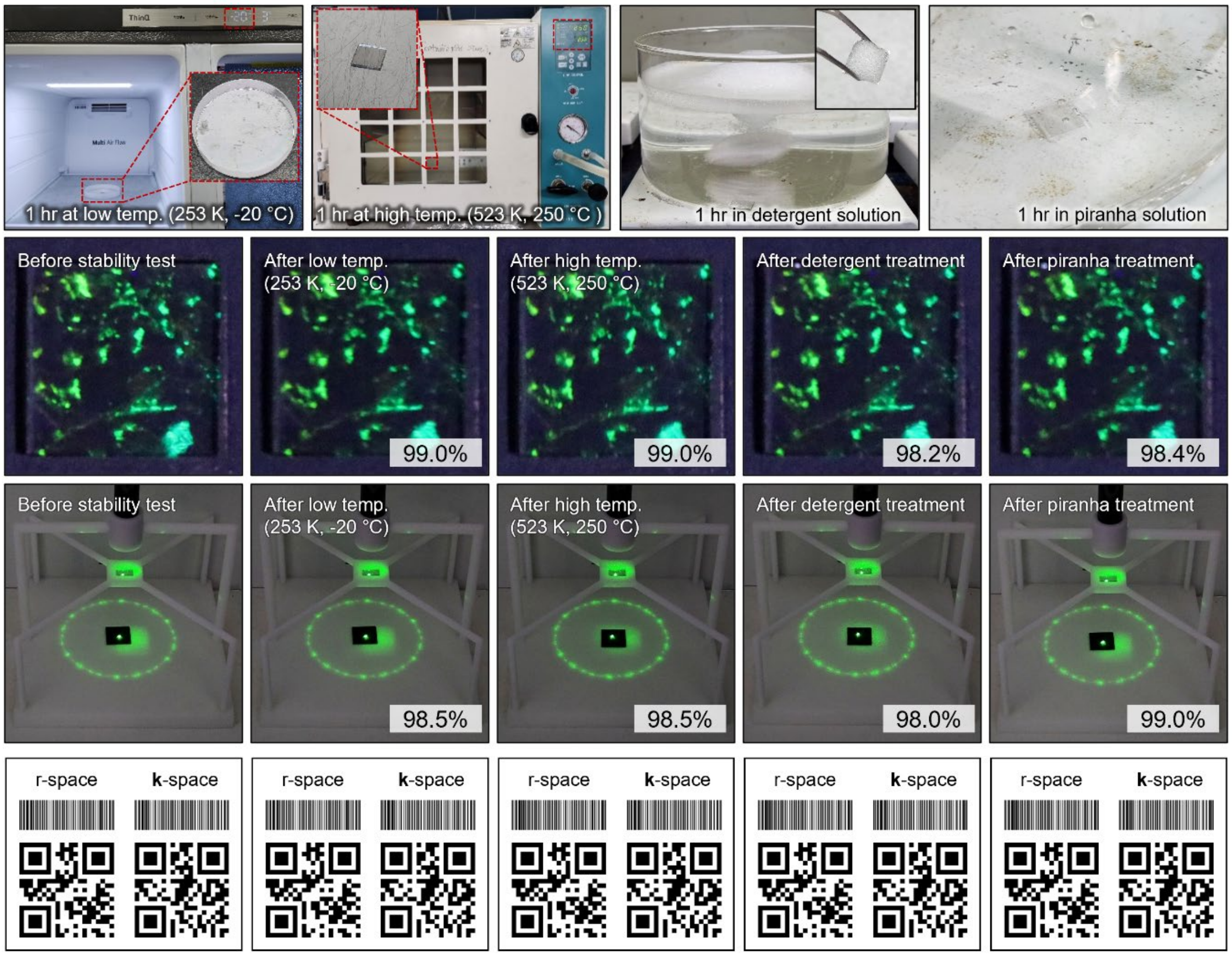

Supplementary Fig. 24 | Thermal and chemical stability tests of the PUF label, and Bragg reflection and diffraction patterns before and after testing. Bit consistency rates are shown in the lower right corner of each image.

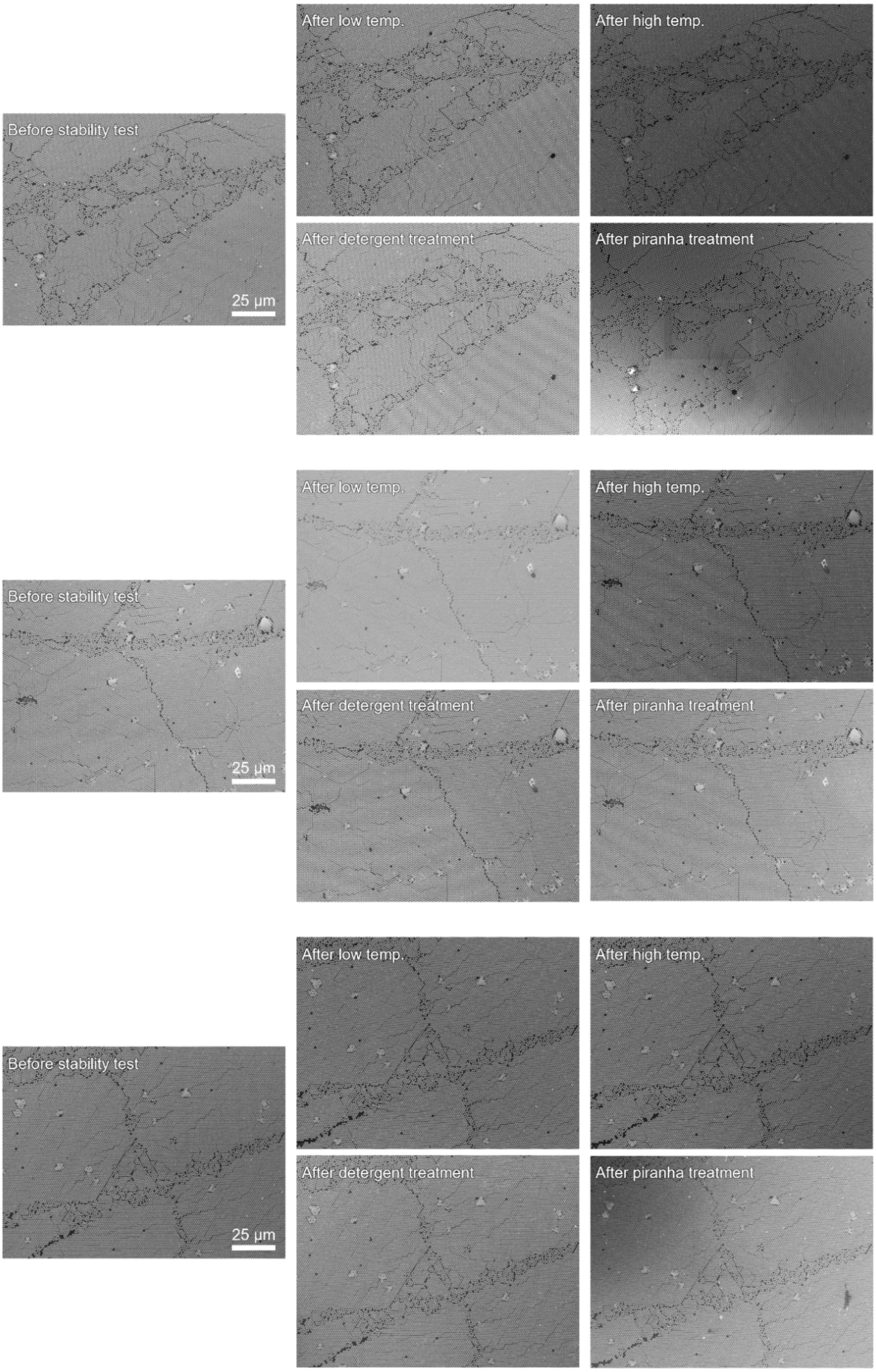

Supplementary Fig. 25 | SEM image of PUF label before and after stability testing.

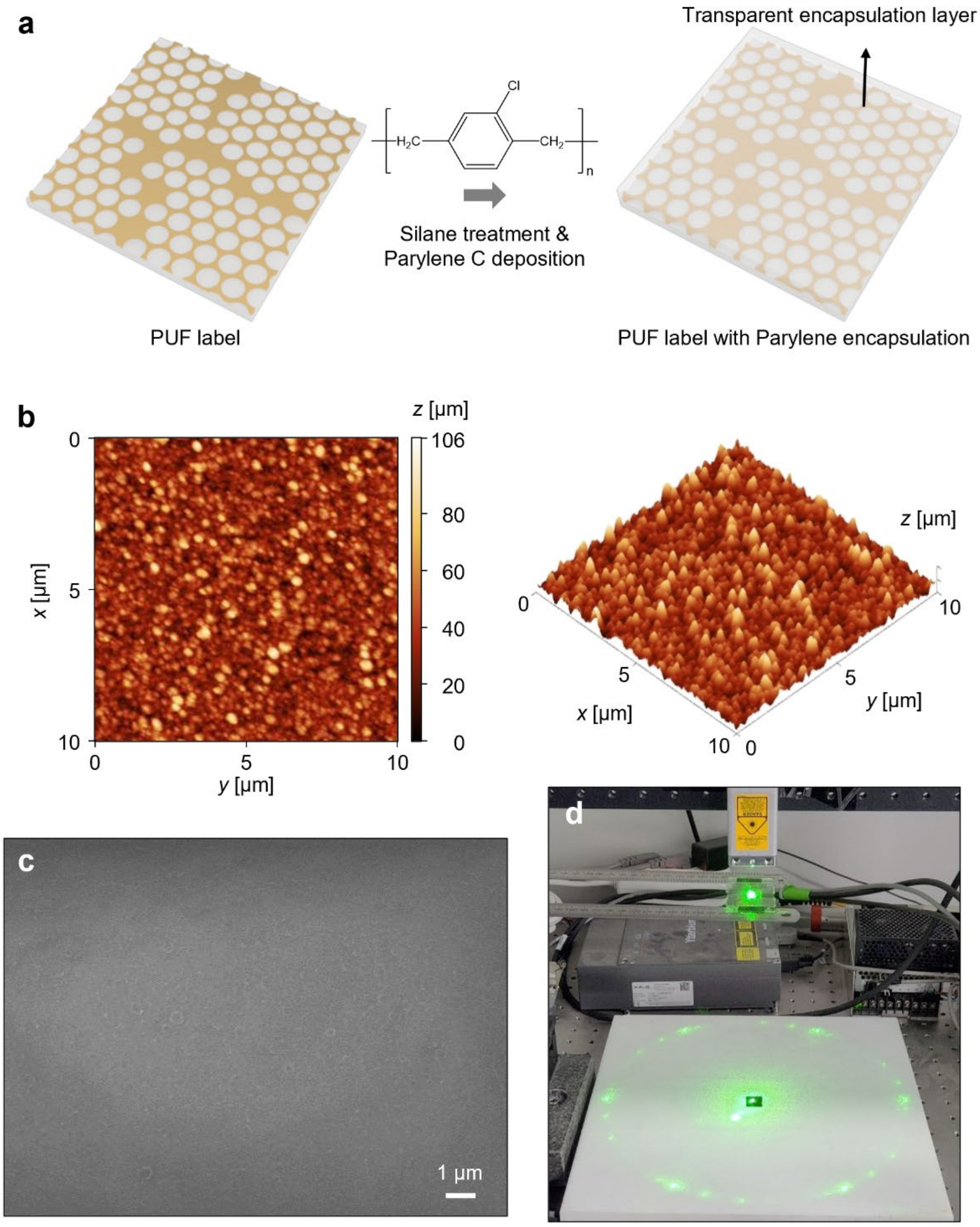

Supplementary Fig. 26 | Parylene encapsulation strategies for pattern replication prevention. a, Schematic illustration of Parylene C deposition on a PUF label. **b,** 2D and 3D AFM images of the Parylene deposited PUF label. **c,** Surface SEM image of the Parylene deposited PUF label. **d,** Diffraction pattern of the Parylene deposited PUF label.

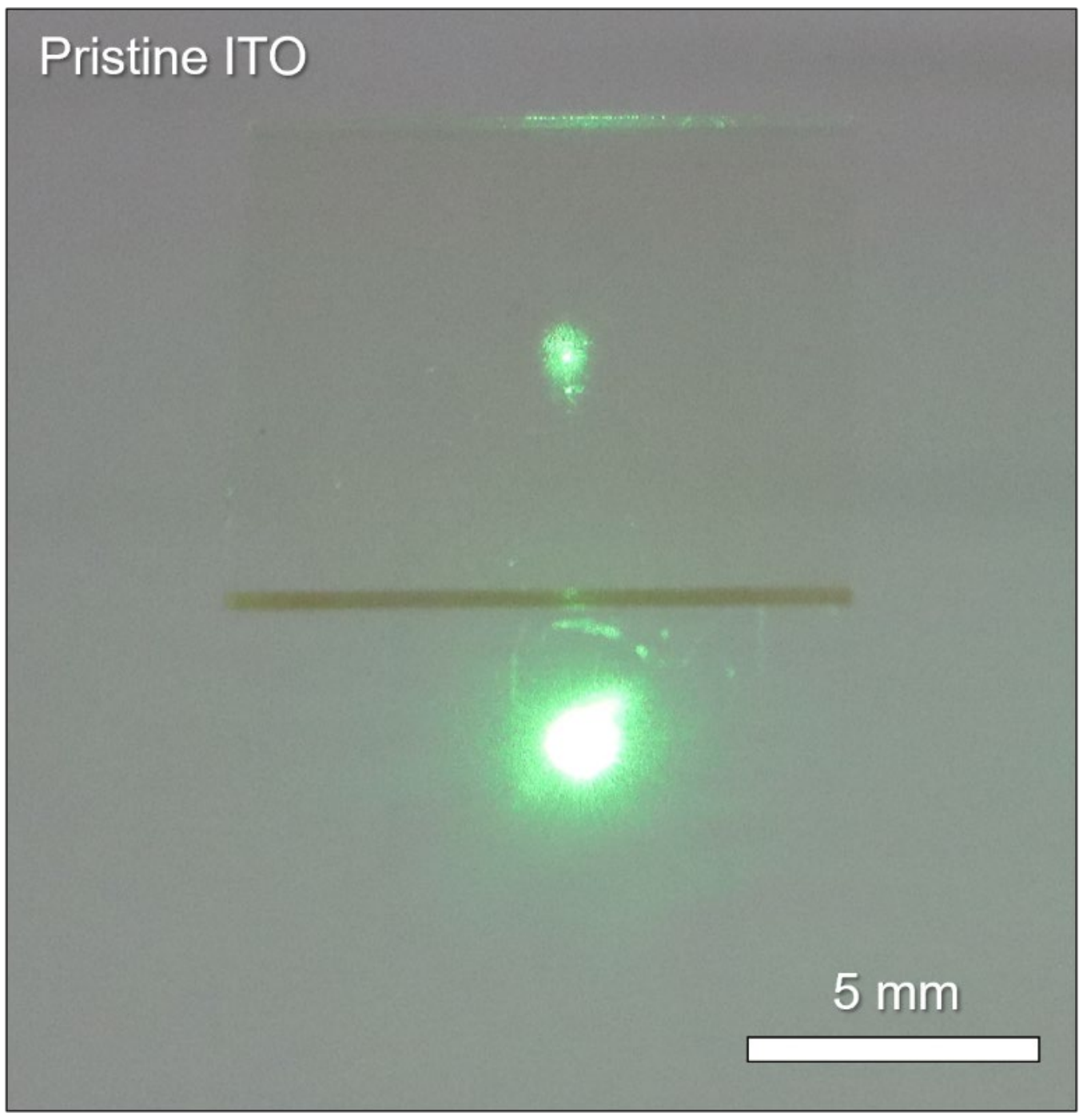

Supplementary Fig. 27 │ Laser irradiation on pristine ITO.

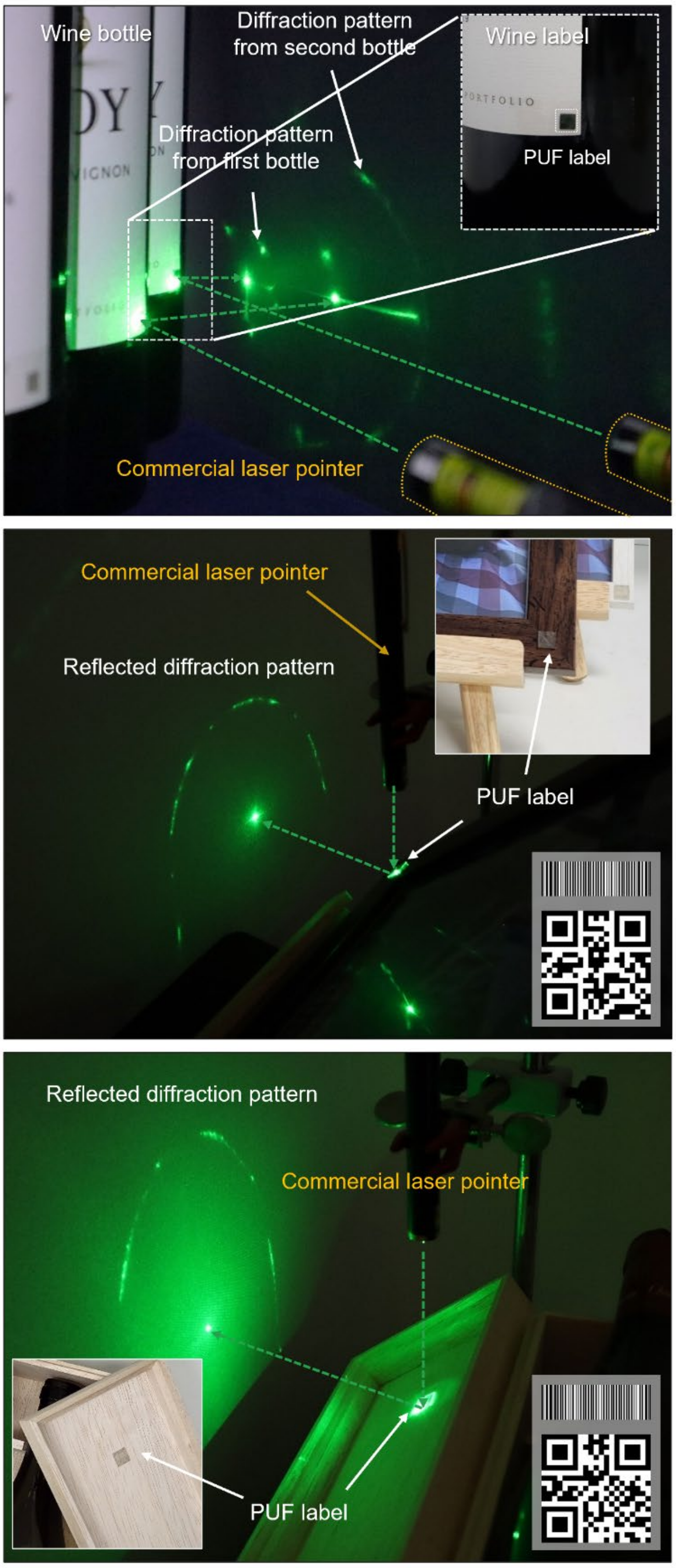

Supplementary Fig. 28 | Practical demonstration of PUF label usage based on reflected diffraction pattern. Implementation of an authentication method using different PUFs attached to liquor bottles, painting frames, and liquor boxes. Authentication is achieved through the reflected diffraction pattern produced by a Cr mesh placed on a SiO_2 or glass substrate.

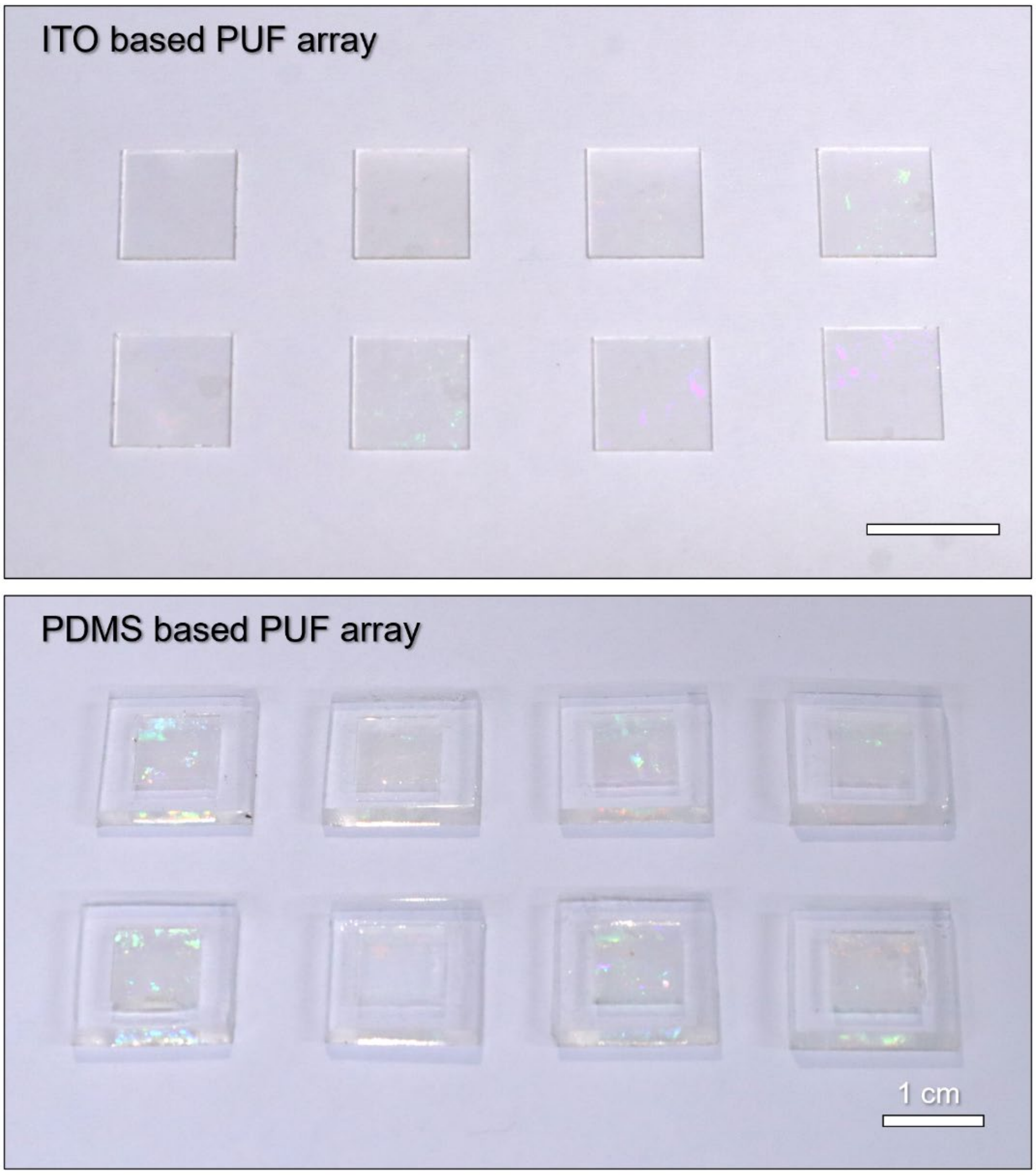

Supplementary Fig. 29 | Digital images of ITO and PDMS based PUF array.

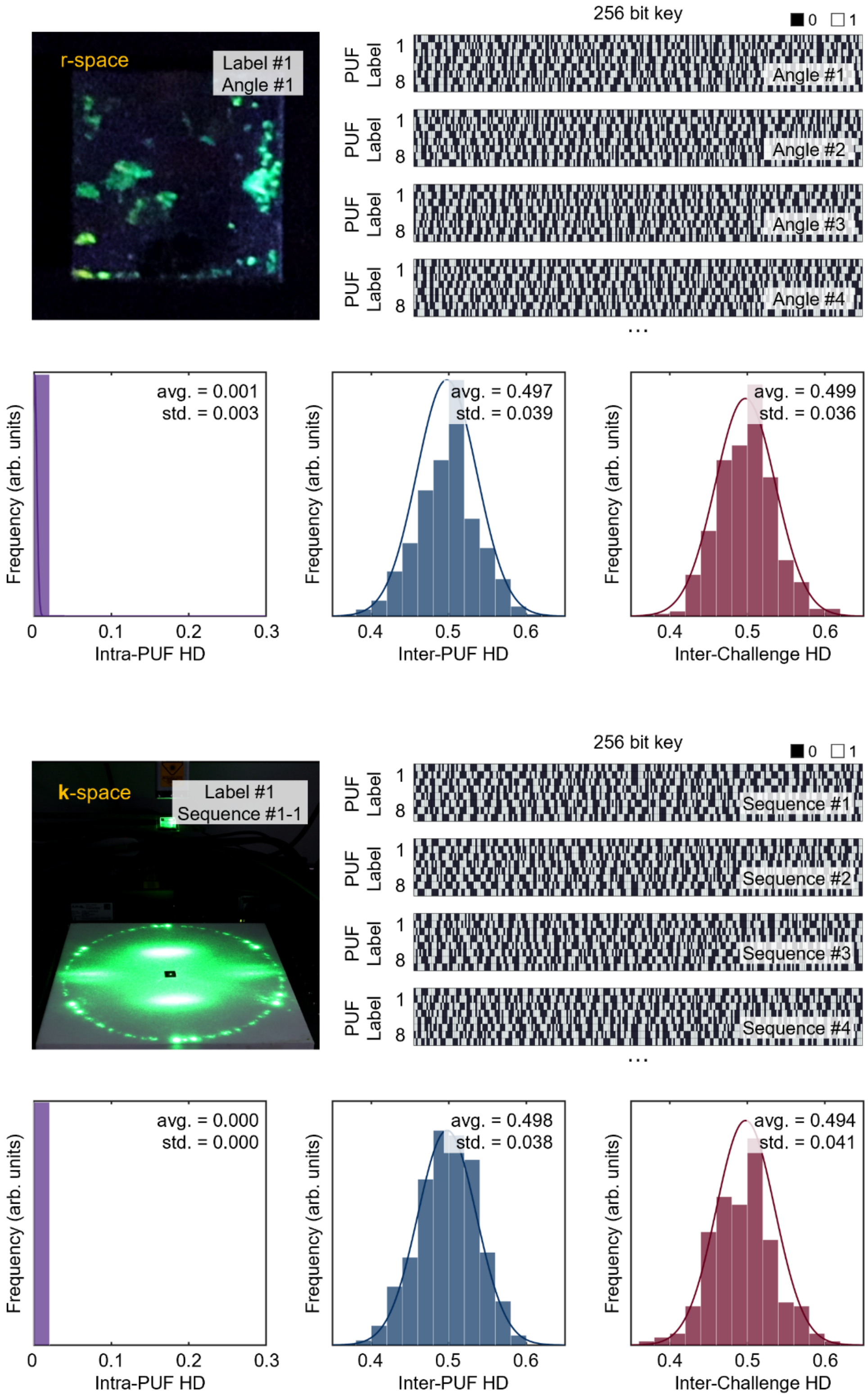

Supplementary Fig. 30 | PDMS-based dual space PUF characteristics in r-space and k-space. Response patterns (left), extracted 256-bit keys (right), and Hamming distance (HD) distributions showing intra-PUF, inter-PUF, and inter-challenge (bottom) for r-space (top) and k-space (bottom) configurations.

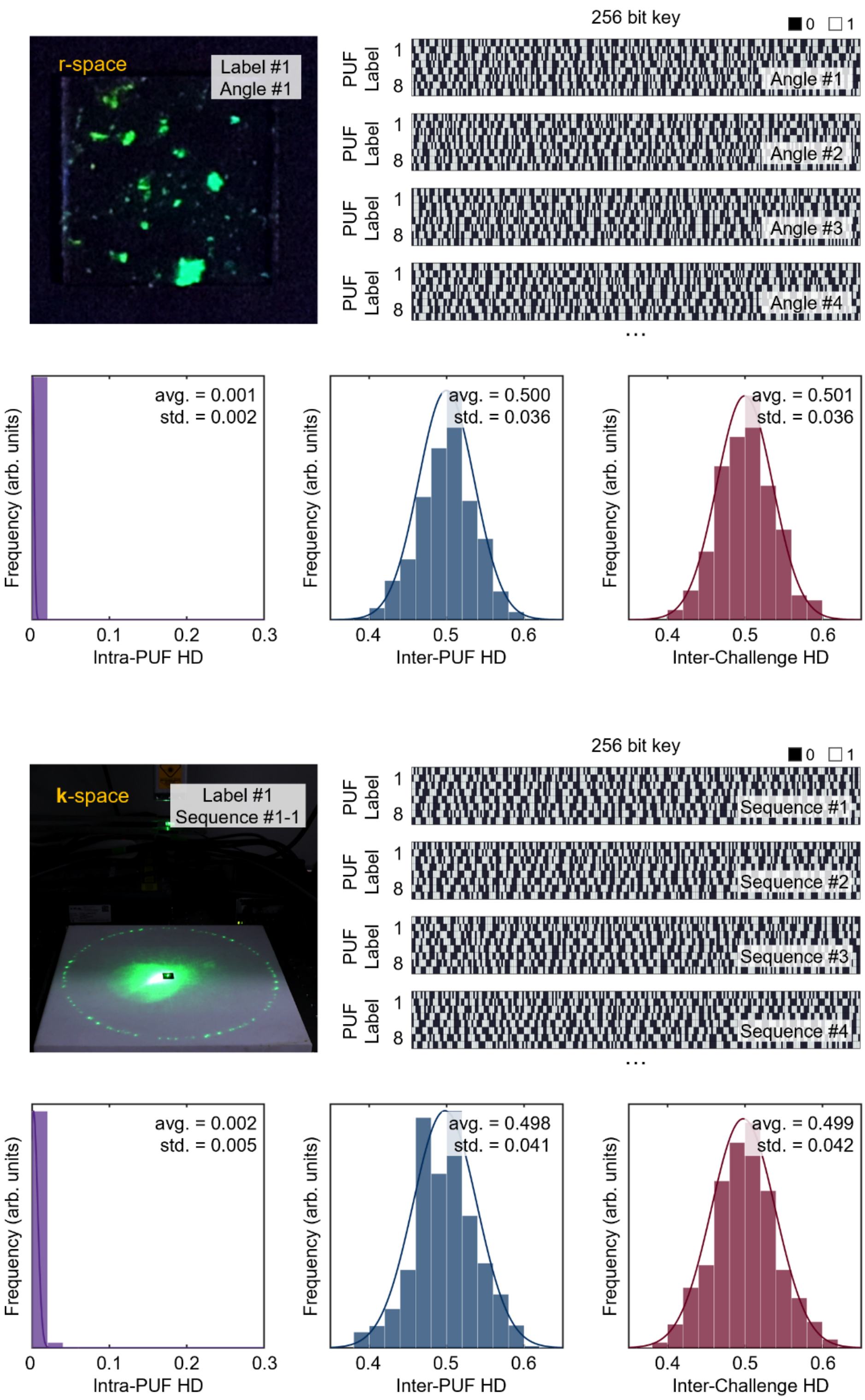

Supplementary Fig. 31 | ITO-based dual space PUF characteristics in r-space and k-space. Response patterns (left), extracted 256-bit keys (right), and Hamming distance (HD) distributions showing intra-PUF, inter-PUF, and inter-challenge (bottom) for r-space (top) and k-space (bottom) configurations.

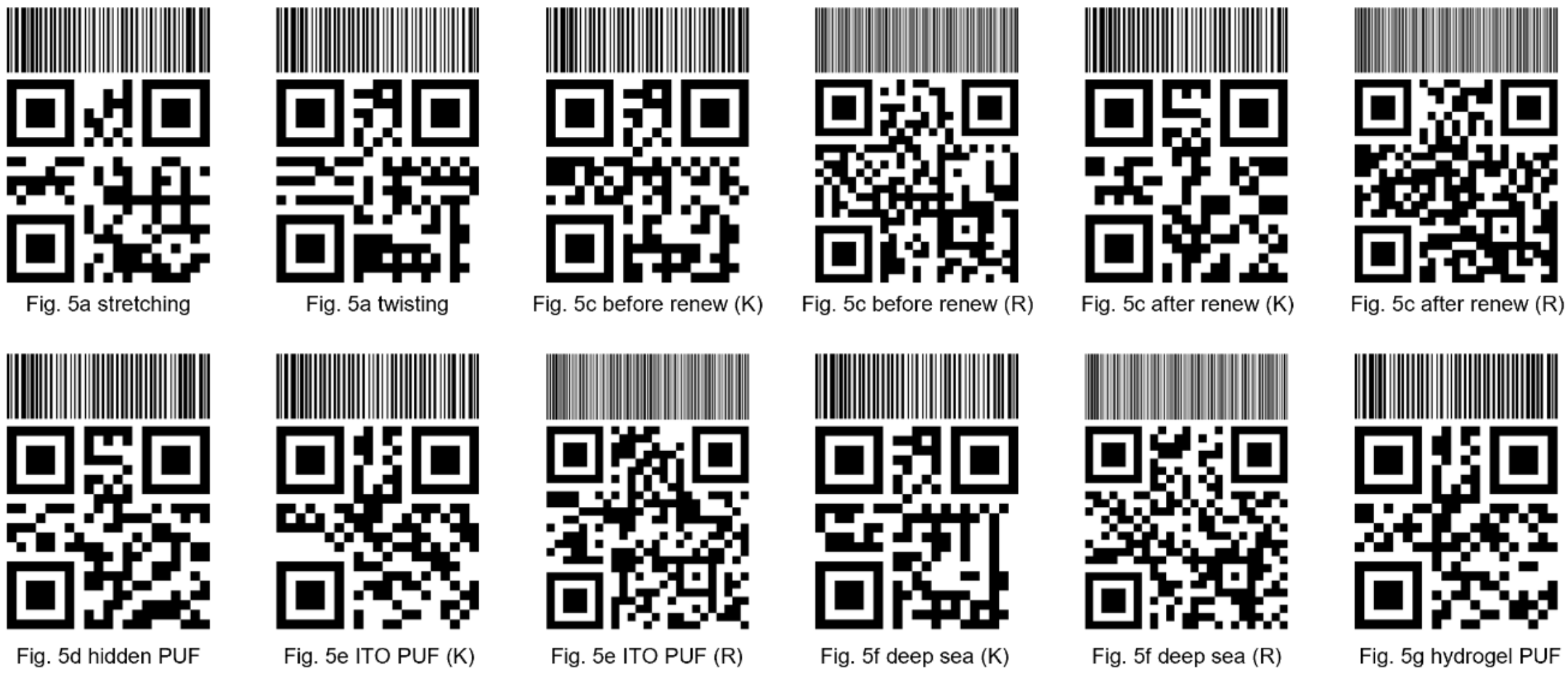

Supplementary Fig. 32 | Corresponding barcode and QR code images of multi-functional PUFs in Fig 5.

	Materials	Vendor	Price	Content	Consumption	Cost
**Fabrication**	PS colloid solution	Thermofisher	575.65 $	10 wt%<br>15ml	~0.1 mg<br>for 4 inch wafer	**3.83 $**<br>for 4 inch wafer
	PS colloid solution (Lab made)	Styrene (Sigma Aldrich)<br>Potassium persulfate (Sigma Aldrich)	602.58 $<br>116.83 $	25 L<br>0.5 kg	16.22 μl<br>for 4 inch wafer<br>6.95 mg<br>for 4 inch wafer	**0.000236 $**<br>for 4 inch wafer<br>**0.000982 $**<br>for 4 inch wafer
	Cr	Kurt J. Lesker	318 $	1 kg	0.28 mg<br>for 4 inch wafer	**0.089 $**<br>for 4 inch wafer
	**Total cost per PUF label:** Commercialized solution (3.83+0.089)/70 = **0.0563 $**<br>Lab made solution (0.000236+0.000982+0.089)/70 = **0.00129 $**					
**Authentication**	ABS-like resin	Anycubic	19.99 $	1 kg	95.2 g	**1.9 $**
	**Total cost: 1.9 $**					

Supplementary Table. 1 │ Calculated fabrication costs of the PUF label and user-kit.
