# Supplementary material for: Dual-space visible light authentication toward high security physical unclonable function
Source: Nat Commun. 2026 Jul 23;17:8967. doi: 10.1038/s41467-026-75781-4 (PMC13503782; doi:10.1038/s41467-026-75781-4)
Supplement: Supplementary file 2 [file 41467_2026_75781_MOESM2_ESM.pdf]

Description of Additional Supplementary Files

Supplementary Video 1: Self-assembly process of PS colloidal polycrystals

Supplementary Video 2: Diffraction-pattern response generation by PUF label scanning
