# Supplementary material for: Dual-space visible light authentication toward high security physical unclonable function
Source: Nat Commun. 2026 Jul 23;17:8967. doi: 10.1038/s41467-026-75781-4 (PMC13503782; doi:10.1038/s41467-026-75781-4)
Supplement: Supplementary file 3 [file 41467_2026_75781_MOESM5_ESM.pdf]

nature portfolio

Peer Review File

Dual-space visible light authentication toward high security physical unclonable function

Corresponding Author: Professor Sang Ouk Kim

This manuscript has been previously reviewed at another journal. This document only contains information relating to versions considered at Nature Communications.

This file contains all reviewer reports in order by version, followed by all author rebuttals in order by version.

Version 0:

Reviewer comments:

Reviewer #1

(Remarks to the Author)
The manuscript reports a dual-space optical physical unclonable functon(PUF) based on polycrystalline colloidal self-assembly, where Bragg reflection in real space and diffraction in reciprocal space are simultaneously exploited for authentication and encryption. It confirms that the polycrystalline multi-scale morphology has been verified more practical than fully randomized disordered assembly for straightforward PUF applications. The manuscript concludes soundly, especially regarding the optical PUF labels based on nanometer-scale self-assembly, which are simple to prepare, easy to authenticate, and highly secure, offering new prospects for artificial information security. I recommend this manuscript to be accepted for publication in Nature Communications. Below are some suggestions that I hope will be helpful:

1. The key point of this work is that the real-space Bragg reflection and reciprocal-space diffraction responses are sufficiently independent to constitute a dual-security authentication mechanism. However, from a Fourier optics perspective, r-space and k-space information are intrinsically correlated. They all originate from the same structure. Is there sufficient data to support independence?
2. The authors attribute the real-space response to “Bragg reflection” from polycrystalline monolayer assemblies. Why azimuthal angle Φ strongly modulates the reflected pattern? Shouldn't this be the result of a multilayered periodic structure?

Reviewer #2

(Remarks to the Author)
The manuscript makes a significant contribution to the field of optical PUFs. The analysis and presentation of the data are thorough and well executed. Collectively, together with the revisions made by the authors, the manuscript in its current form is recommended for publication in Nature Communications.

Reviewer #3

(Remarks to the Author)
Authors address all concerns raised by the reviewers and clearly stated the major advantages of their work in the revision. Therefore, I recommend acceptance for publication without further revision.

Open Access This Peer Review File is licensed under a Creative Commons Attribution 4.0 International License, which permits use, sharing, adaptation, distribution and reproduction in any medium or format, as long as you give appropriate credit to the original author(s) and the source, provide a link to the Creative Commons license, and indicate if changes were made.
In cases where reviewers are anonymous, credit should be given to 'Anonymous Referee' and the source.
The images or other third party material in this Peer Review File are included in the article's Creative Commons license, unless indicated otherwise in a credit line to the material. If material is not included in the article's Creative Commons license and your intended use is not permitted by statutory regulation or exceeds the permitted use, you will need to obtain permission directly from the copyright holder.
To view a copy of this license, visit https://creativecommons.org/licenses/by/4.0/

<u>Response to Reviewer's Comments</u>

(Reviewer's comment in plain text, Author's response in blue letter)

Manuscript number: NCOMMS-26-035582-T

Title: Dual-space visible light authentication towards high-security physical unclonable function

Reviewer #1 (Remarks to the Author):

The manuscript reports a dual-space optical physical unclonable functon(PUF) based on polycrystalline colloidal self-assembly, where Bragg reflection in real space and diffraction in reciprocal space are simultaneously exploited for authentication and encryption. It confirms that the polycrystalline multi-scale morphology has been verified more practical than fully randomized disordered assembly for straightforward PUF applications. The manuscript concludes soundly, especially regarding the optical PUF labels based on nanometer-scale self-assembly, which are simple to prepare, easy to authenticate, and highly secure, offering new prospects for artificial information security. I recommend this manuscript to be accepted for publication in Nature Communications. Below are some suggestions that I hope will be helpful:

Author's reply: We appreciate the reviewer's positive evaluation of our revised manuscript. We hope that our response to the additional questions has satisfactorily addressed the reviewer's concerns.

1. The key point of this work is that the real-space Bragg reflection and reciprocal-space diffraction responses are sufficiently independent to constitute a dual-security authentication mechanism. However, from a Fourier optics perspective, r-space and k-space information are intrinsically correlated. They all originate from the same structure. Is there sufficient data to support independence?

Author's reply: We appreciate the reviewer's valuable comment. As the reviewer correctly pointed out, the Bragg reflection in real space and the diffraction pattern in reciprocal space are not strictly independent in a mathematical Fourier-optics sense, because both responses originate from the same underlying colloidal assembly, represented as the virtual three-dimensional space in Fig. 1h. Therefore, a certain degree of intrinsic correlation may exist between the two optical readout modes.

However, as described in the manuscript, the independence of the two signals is discussed from the viewpoint of encryption and authentication, rather than strict mathematical independence. In this context, "independence" means that the response obtained from one readout mode cannot practically provide sufficient information to infer, predict, or reconstruct the response from the other mode.

This point is supported by Supplementary Figs. 4 and 5. These analyses show that a single diffraction pattern can correspond to numerous possible Bragg reflection patterns, and conversely, a single Bragg reflection pattern can correspond to numerous possible diffraction patterns. This is because each optical response extracts only a highly incomplete subset of the full structural information in the virtual three-dimensional space. The real-space Bragg reflection mainly reflects the spatial distribution of grains satisfying a given illumination condition, whereas the reciprocal-space diffraction response represents the local distribution of lattice orientations within the laser-illuminated region.

Therefore, even if one optical response is disclosed, the counterpart response is not uniquely determined. Furthermore, even when partial information from both modes is considered, the possible colloidal configurations capable of producing the observed responses remain extremely large because the detailed spatial arrangement, grain-boundary configuration, and nanoscale defects are not uniquely determined by the extracted optical signals.

Accordingly, the two responses are not fundamentally independent in the strict Fourier-optics sense, but are sufficiently decoupled and mutually non-reconstructable from the encryption and authentication viewpoint. This practical independence supports the use of real-space Bragg reflection and reciprocal-space diffraction as a dual-security authentication mechanism.

2. The authors attribute the real-space response to “Bragg reflection” from polycrystalline monolayer assemblies. Why azimuthal angle Φ strongly modulates the reflected pattern? Shouldn't this be the result of a multilayered periodic structure?

Author’s reply: We appreciate the reviewer’s valuable comment. There are two types of Bragg reflection that can arise from colloidal assembly structures. First, when colloids are assembled into a three-dimensional structure, a *d*-spacing exists along the thickness direction. This gives rise to a photonic stop band that reflects wavelengths satisfying Bragg’s law. As a result, the entire PUF label uniformly reflects only the wavelength corresponding to the photonic stop band and exhibits a uniform color regardless of the azimuthal angle, even if the structure is polycrystalline.

Second, as in our structure, when colloids are assembled as a monolayer, there is no *d*-spacing along the thickness direction. Instead, the *d*-spacing corresponds to the periodicity of the colloids in the in-plane direction. Light scattered from this in-plane periodic pattern undergoes

constructive interference, and the wavelength that satisfies the constructive interference condition is determined by the angular difference between the incident light and the crystal orientation, as shown in Supplementary Fig. 8. However, except when the incident light is aligned with the crystal orientation, constructive interference occurs only at wavelengths far beyond the visible range. Therefore, Bragg reflection occurs only in selected local regions.

For this reason, to obtain an azimuthal-angle-sensitive signal as demonstrated in our manuscript, the system must be composed of a monolayer and must have a polycrystalline structure, as in our system. A detailed discussion of this point can be found in Supplementary Fig. 1.

Reviewer #2 (Remarks to the Author):

The manuscript makes a significant contribution to the field of optical PUFs. The analysis and presentation of the data are thorough and well executed. Collectively, together with the revisions made by the authors, the manuscript in its current form is recommended for publication in Nature Communications.

Author's reply: We sincerely thank the reviewer for the positive evaluation of our manuscript. We hope that our study will contribute meaningfully to the advancement of colloidal self-assembly and PUF-related research.

Reviewer #3 (Remarks to the Author):

Authors address all concerns raised by the reviewers and clearly stated the major advantages of their work in the revision. Therefore, I recommend acceptance for publication without further revision.

Author's reply: We sincerely thank the reviewer for the positive evaluation of our manuscript. We hope that our study will contribute meaningfully to the advancement of colloidal self-assembly and PUF-related research.
